# Task-induced 1/f slope modulation as a paradigm-independent marker of cognitive control in multiple sclerosis

**DOI:** 10.1162/IMAG.a.973

**Published:** 2025-10-30

**Authors:** Fahimeh Akbarian, Máté Gyurkovics, Marie B. D’hooghe, Miguel D’haeseleer, Guy Nagels, Jeroen Van Schependom

**Affiliations:** Department of Electronics and Informatics (ETRO), Vrije Universiteit Brussel, Brussels, Belgium; AIMS lab, Vrije Universiteit Brussel, Center for Neurosciences, Brussels, Belgium; School of Psychology, University of East Anglia, Norwich, United Kingdom; National MS Center Melsbroek, Melsbroek, Belgium; Center for Neurosciences, Vrije Universiteit Brussel, Brussels, Belgium; UZ Brussel, Department of Neurology, Brussels, Belgium; St Edmund Hall, University of Oxford, Oxford, United Kingdom

**Keywords:** aperiodic 1/f slope, multiple sclerosis, magnetoencephalography (MEG), excitation/inhibition (E/I) balance, aperiodic activity, auditory oddball paradigm

## Abstract

Multiple sclerosis (MS) is a chronic neuro-degenerative and inflammatory disease causing motor, sensory, and cognitive deficits, including impairments in working memory and attention. These cognitive deficits may arise from an imbalance between excitatory and inhibitory neural activity due to synaptic loss. Recent studies suggest that the aperiodic 1/f slope, a neural marker reflecting excitation/inhibition (E/I) balance, could serve as a biomarker for cognitive control. This study examines 1/f slope modulation during cognitive tasks in people with MS and healthy controls to investigate its potential as a paradigm-independent marker of cognitive control. We analyzed the Magnetoencephalography (MEG) data collected from 126 participants: 44 healthy controls (HCs), 61 people with MS not treated with benzodiazepines (pwMS(BZDn)), and 21 pwMS treated with benzodiazepines (pwMS(BZDp)). Participants performed an auditory oddball task and a visual-verbal n-back working memory task. After preprocessing MEG data, we used the specparam (formerly FOOOF) algorithm to extract the aperiodic 1/f slope from power spectral densities across 42 cortical parcels. Through this analysis, we observed significant steepening in the 1/f slope following stimulus onset for all stimulus types, with non-standard stimuli (targets and distractors) producing more pronounced effect (i.e., steeper slopes following stimulus onset). Compared to HCs, people with MS treated with benzodiazepines showed attenuated slope steepening in response to distractor stimuli, consistent with benzodiazepine-related impairments in inhibitory control. Moreover, unlike HCs, pwMS exhibited less steepening of 1/f slope response to distractors versus targets, indicating deficient phasic inhibition. Finally, in both HCs and pwMS(BZDn), the 1/f slope modulation was positively correlated across the auditory oddball and n-back tasks, pointing to a consistent, paradigm-independent neural mechanism. Taken together, these findings demonstrate that the aperiodic 1/f slope is a sensitive, paradigm-independent marker of cognitive control and E/I balance. The attenuated slope steepening in response to distractors in pwMS highlights disruptions in inhibitory neural processes underlying their cognitive deficits. These findings underscore the value of aperiodic spectral measures to deepen our understanding of cognitive impairments in MS.

## 1 Introduction

Multiple sclerosis (MS) is a chronic autoimmune disorder characterized by widespread demyelination and neurodegeneration within the central nervous system. Beyond motor and sensory deficits, MS is frequently associated with significant cognitive impairments, particularly in working memory (WM), attention, information processing speed, and executive functions (Benedict et al., 2004; Rao et al., 1991). Despite extensive research, the neural mechanisms underlying cognitive impairments in MS remain poorly understood.

One characteristic of MS pathology is synaptic loss, particularly affecting inhibitory synapses, leading to disruption of the balance between excitation and inhibition (E/I) in neural circuits (Huiskamp et al., 2022; Zoupi et al., 2021). This imbalance leads to reduced network stability and impaired cognitive control, which may underline the observed cognitive deficits in people with MS (pwMS) (Huiskamp et al., 2022). One possible approach to understanding the neural substrates of cognitive impairments in MS is to investigate the imbalance in the dynamic interaction between excitatory and inhibitory neuronal circuits.

Traditional neurophysiological studies aimed at understanding cognitive impairment in MS have long focused on oscillatory neural activity by analyzing rhythms within specific frequency bands (Keune et al., 2017; Khan et al., 2021; Simon et al., 2023). While these oscillatory measures provide valuable insights, they only capture one aspect of the rich dynamics of neural activity. In recent years, there has been growing interest in the non-oscillatory or aperiodic component of the neural power spectrum, often modelled as 1/f activity, as a complementary approach to understanding neural function. The 1/f activity captures the broadband shape of the power spectrum where power typically decreases as a function of frequency (or f) according to a power-law distribution, typically approximated by 1/f^x^ (Donoghue et al., 2020). Unlike narrowband oscillatory measures, the aperiodic 1/f component reflects scale-free properties across a wide frequency range, offering a window into the global state of neural excitability and the balance between excitation and inhibition (Gao et al., 2017; He et al., 2010). Recent theoretical and methodological advances have further bolstered the evidence supporting its functional relevance in elucidating brain dynamics and human behavior (Donoghue et al., 2020; Gyurkovics et al., 2022; Kałamała et al., 2023; Voytek et al., 2015; Waschke et al., 2021).

Central to this approach is the 1/f slope, or exponent, which quantifies the rate at which power declines with increasing frequency within the power spectral density of neural data. Evidence suggests that the 1/f slope serves as an index of E/I balance in the brain (Gao et al., 2017). A steeper slope indicates a higher level of inhibition, while a flatter slope indicates increased excitation (Akbarian et al., 2023; Chini et al., 2022; Gao et al., 2017). Importantly, this metric is not merely a reflection of noise in neural activity but is associated with cognitive functioning. Task-induced changes in the 1/f slope have been linked to cognitive demands such as attention and working memory load, highlighting its role as an index of cognitive control (Akbarian et al., 2024; Gyurkovics et al., 2022; Lu et al., 2024; Waschke et al., 2021). Therefore, understanding how the 1/f slope adapts to cognitive demand provides valuable insights into the neural substrates of cognition and their disruption in MS.

In this study, we first explore event-related 1/f slope modulation during an auditory oddball task in pwMS and healthy controls (HCs). The auditory oddball task probes selective attention by requiring participants to discriminate between standard and deviant (target/distractor) stimuli (Squires et al., 1975). Based on previous findings linking steeper spectral slopes to increased cognitive demands (Gyurkovics et al., 2022; Lu et al., 2024), we hypothesize that the 1/f slope modulation (post-event steepening) will be more pronounced for non-standard (target/distractor) stimuli than standard stimuli. We also hypothesize that pwMS will exhibit lower task-related 1/f slope steepening than HCs during distractor trials, as we previously observed during a working memory task, potentially due to a disruption in their cortical E/I balance (Akbarian et al., 2024).

We further aim to investigate whether the 1/f slope modulation remains consistent across different tasks. Specifically, we examine whether the modulation of the 1/f slope observed during the auditory oddball task correlates with that observed in a visual-verbal n-back task, as suggested by our previous findings (Akbarian et al., 2024). The n-back task, a widely used measure of working memory, involves monitoring and updating information with varying levels of difficulty (0-back, 1-back, 2-back) (Costers et al., 2020). By assessing 1/f slope modulation in both tasks, we aim to determine whether these changes are task-specific or indicative of a shared, paradigm-independent cognitive control mechanism. In light of recent findings showing that aperiodic modulations are sensitive to multiple types of cognitive demand (working memory, task switching, and inhibitory control (Lu et al., 2024)), we predicted results to be in favor of the latter alternative. Furthermore, demonstrating correlations across the two tasks would support the notion that the 1/f slope reflects a shared neurophysiological mechanism potentially a trait-like indicator of top-down control capacity rather than being solely task-specific.

## 2 Methods

### 2.1 Participants

MEG data were collected from 126 participants during an auditory oddball task, including 21 people with MS (pwMS) receiving benzodiazepine treatment (pwMS(BZDp)), 61 pwMS not receiving benzodiazepines (pwMS(BZDn)), and 44 healthy controls (HCs). Table 1 presents a detailed description of subjects. Additionally, 101 of these participants (34 HCs, 17 pwMS(BZDp), 50 pwMS(BZDn)) also completed a visual-verbal n-back task. Given our recent findings (Akbarian et al., 2023) that benzodiazepines influence the 1/f spectral slope, we categorized the pwMS group based on their benzodiazepine treatment status. The participants were between 18 and 65 years old (mean = 47.85, SD = 10.64). All pwMS were recruited from the National MS Center Melsbroek and were diagnosed with multiple sclerosis according to the revised McDonald’s criteria (Polman et al., 2011). They had an Expanded Disability Status Scale (EDSS) score of 6 or lower (Kurtzke, 1983) to ensure the feasibility of patient participation in data acquisition. Exclusion criteria included a history of relapses or corticosteroid treatment within the previous 6 weeks, as well as the presence of a pacemaker, dental wires, major psychiatric disorders, or epilepsy. All participants provided written informed consent before participation. The study received ethical approval from the local ethics committees of the University Hospital Brussels (Commissie Medische Ethiek UZ Brussel, B.U.N. 143201423263, 2015/11) and the National MS Center Melsbroek (2015-02-12).

### 2.2 Data acquisition

The MEG data were collected at the CUB Hôpital Erasme (Brussels, Belgium) on an Elekta Neuromag Vectorview scanner (Elekta Oy, Helsinki, Finland) for the first 30 pwMS and 14 healthy subjects and the rest of subjects were scanned using an upgraded scanner, Elekta Neuroimage Triux scanner (MEGIN, Croton Healthcare, Helsinki, Finland). Both MEG scanners used a sensor layout with 102 triple sensors, each consisting of one magnetometer and two orthogonal planar gradiometers and were placed in a lightweight magnetically shielded room (MaxshieldTM, Elekta Oy, Helsinki, Finland). MEG data were recorded during the auditory oddball and n-back task data. Structural MRI scans were obtained using a 3T Philips MRI system with a T1-weighted sequence at of 1 × 1 × 1 mm³ resolution. The acquisition parameters included a repetition time (TR) of 4.93 ms, a flip angle (FA) of 8°, a field of view (FOV) of 230 × 230 mm², and 310 sagittal slices, achieving a voxel resolution of 0.53 × 0.53 × 0.53 mm³.

### 2.3 MEG processing and parcellation

MEG signals were recorded with a 0.1–330 Hz passband filter at 1 kHz sampling rate. The preprocessing of MEG data began with applying the temporal extension of the signal space separation algorithm (MaxFilter™, Elekta Oy, Helsinki, Finland, version 2.2, default parameters) to eliminate external interferences and correct for head movements (Taulu et al., 2005). Subsequent preprocessing was conducted using the Oxford Software Library (OSL) pipeline, which integrates tools from FSL, SPM12 (Wellcome Trust Centre for Neuroimaging, University College London), and FieldTrip (Oostenveld et al., 2011).

First, we applied a finite impulse response (FIR) anti-aliasing low-pass filter with a cut-off frequency at 125 Hz, then the data were downsampled to 250 Hz. Coregistration with the subject’s T1-weighted MRI was performed automatically using OSL’s RHINO algorithm (https://github.com/OHBA-analysis). Head shape points were aligned with the scalp surface extracted via FSL’s BETSURF and FLIRT (Jenikson, 2005; S. M. Smith, 2002) and subsequently transformed into the common MNI152 space (Mazziotta et al., 1995). The data were then band-pass filtered between 0.1 and 70 Hz, and a 5th-order Butterworth notch filter (49.5–50.5 Hz) was applied to remove power line noise.

To address artefacts, a semi-automated independent component analysis (ICA) was performed to visually identify and remove ocular and cardiac artefacts, based on the correlation of component time series with electrooculogram (EOG) and electrocardiogram (ECG) signals, respectively. For source reconstruction, we projected sensor‐level data into source‐space using a linearly constrained minimum variance (LCMV) beamformer enhanced with Bayesian PCA to stabilize high‐dimensional covariance estimates and suppress sensor noise as developed by Woolrich et al. (2011). This method automatically determines an optimal low‐dimensional subspace for the data covariance, improving signal‐to‐noise ratio before beamforming.

The forward model was built on an isotropic single‐shell volume conductor in MNI space, discretized on an 8 mm grid of dipoles. To align template anatomy with the MEG sensor positions, fiducial points (nasion and preauriculars) were co‐registered to each participant’s head position data. From this, we computed the lead‐field matrix for each grid point using FieldTrip (Oostenveld et al., 2011), resulting the mapping from a dipole at each location to the MEG sensors.

The source-reconstructed signals were then parcelled using a predefined atlas consisting of 42 cortical parcels (Vidaurre et al., 2018). Within each parcel, the first principal component (PC1) of the time series was extracted and used as the representative signal. Across parcels and subjects, this component captured 65.0 % ± 10.6 % (mean ± SD) of the within‐parcel variance. The distributions of variance explained by PC1 across subjects and parcels are reported in Figure S1–S3 and Table S1. The parcellation atlas was designed to cover the entire cortex, excluding subcortical regions (Van Schependom et al., 2019; Vidaurre et al., 2018).

### 2.4 Auditory oddball task

The subjects were presented with a series of 400 auditory tones over a total test duration of 8 min and 20 s. Eighty percent of the tones were categorized as frequent tones (1000 Hz), 10% as target tones (1500 Hz), and 10% as distractor tones (500 Hz). Subjects were instructed to press a button upon detecting a high-pitched target tone amidst the series of standard tones. The interstimulus interval was randomized between 1 and 1.5 s. Accuracy was defined as the proportion of correct responses (either correct detections or correct rejections) relative to the total number of trials.

### 2.5 Visual-verbal n-back task

During the MEG acquisition, participants were asked to perform an n-back task (Costers et al., 2020) with three conditions or levels of working memory load (0, 1 and 2-back). The n-back task includes trials that require a response and those that do not, referred to as “target” trials and “distractor” trials, respectively. The examiner instructed participants to press a button with their right hand when the letter displayed on the screen was the letter X (0-back condition), the same letter as the one before (1-back condition), or the same letter as two letters before (2-back condition). There were 240 stimuli, with 25, 23, and 28 target trials per condition (0-back, 1-back, 2-back), respectively.

### 2.6 Power spectral analysis and estimation of aperiodic components

We calculated the power spectral density (PSD) for each trial and parcel using SciPy’s default Welch function, following the method described in (Akbarian et al., 2024) to extract the 1/f slope modulation. Next, we applied the specparam (formerly “Fitting Oscillations and One-Over F” (FOOOF)) algorithm (Donoghue et al., 2020) to estimate the 1/f exponent that quantifies the steepness of the 1/f slope. This algorithm iteratively fits Gaussian functions to the periodic components of the PSD and subtracts them, thereby isolating the aperiodic 1/f component. The fitting was performed over 4 Hz to 44 Hz frequency range. The fitting was performed over 4 Hz to 44 Hz frequency range. With the 500 ms epochs (frequency resolution equal to 2 Hz), bins below 4 Hz might have low signal-to-noise ratio and be dominated by drift, and bins above 44 Hz lie close to 50 Hz power line noise notch filter; thus, we restricted the fitting frequency range to 4–44 Hz to ensure capturing clean neuronal background activity.

In the auditory oddball paradigm, each trial provided 1 s of data (0.5 s before and 0.5 s after the stimulus), while in the n-back paradigm, we used 1-s windows both before and after the stimulus onset. First, we excluded trials where the subject had pressed the button on the immediately preceding trial, to eliminate any influence of button presses on the pre-stimulus time window. Then, power spectra densities were computed separately for 42 brain parcels for each subject, and the corresponding 1/f exponents (or slopes) and intercepts (offsets) were extracted using the specparam algorithm.

To adjust for event-related fields (ERFs) during the post-stimulus period, we implemented the method described by Gyurkovics et al. (2022). First, we measured the power spectra from averaged time-domain responses (i.e., the ERFs) for each stimulus type, subject, brain parcel, and time window. These ERF spectra were then subtracted from the total power spectra (i.e., the average of single-trial spectra), effectively isolating induced activity by removing the ERF contribution. We applied the same procedure to correct for ERF-related effects in the power spectra from the n-back task data.

**Fig. 1.**
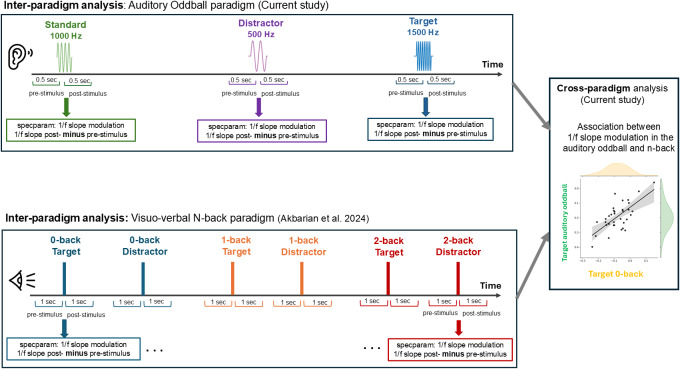
Study pipeline. Top panel: In the current study, MEG data were collected during an auditory oddball task consisting of standard (1000 Hz), distractor (500 Hz), and target (1500 Hz) tones. For each stimulus type, the 1/f slope modulation was computed as the difference between post- and pre-stimulus periods using the specparam algorithm. Bottom panel: Data from a previously published MEG study (Akbarian et al., 2024) with the same individuals using a visuo-verbal n-back task (0-back, 1-back, and 2-back conditions with target and distractor trials) were reanalyzed using the same 1/f slope modulation approach employed in the auditory oddball analysis. Right panel: A cross-paradigm correlation was performed on the findings from auditory oddball and n-back tasks to assess individual-level consistency in 1/f slope modulation between tasks. Each dot in the scatter plot represents one participant’s 1/f slope modulation value in the two tasks (x-axis: target trial in 0-back condition, y-axis: target trial in auditory oddball).

Figure 1 presents the study pipeline.

### 2.7 Neuropsychological assessment

Neuropsychological tests were performed on the same day as the MEG recording for all participants. The test battery included the Symbol Digit Modalities Test (SDMT; A. Smith, 1968) to evaluate information processing speed, the Dutch version of the California Verbal Learning Test (CVLT-II; Strober et al., 2009), the Dutch version: VGLT to assess verbal memory. Verbal fluency was measured by the Controlled Oral Word Association Test (COWAT; Ruff et al., 1996), and spatial memory was evaluated using the Brief Visuospatial Memory Test (Revised; BVMT-R; Benedict et al., 1996). Additionally, Fatigue was assessed by the Fatigue Scale for Motor and Cognitive Function (FSMC; Penner et al., 2009) and depression by Beck’s Depression Inventory (BDI; Beck, 1961).

### 2.8 Statistics

Normality of the 1/f slope values was assessed using the Shapiro–Wilk test (Shapiro & Wilk, 1965) prior to conducting any group-level comparisons. First, we tested each time window (pre- and post-stimulus) × group × parcel combination, resulting in 252 total combinations. Of these, 69 (27.4%) for standard trials, 33 (13.1%) for target trials, and 43 (17.1%) for distractor trials failed the normality criterion (p < 0.05). We also assessed normality for each group × parcel combination of post-minus-pre slope modulation values (126 combinations total); 23 (18.3%) for standard trials, 9 (7.1%) for target trials, and 24 (19.0%) for distractor trials failed the normality test. These proportions exceed the 5% expected false positive rate. Therefore, all subsequent statistical analyses were performed using non-parametric methods. Paired comparisons were assessed using the Wilcoxon signed-rank test, while non-paired comparisons were analyzed using the Mann-Whitney U test. To account for multiple comparisons, Benjamini-Yekutieli false discovery rate (FDR) correction (Benjamini & Hochberg, 1995) was applied to adjust p-values, with the false positive rate set to 5%. Effect sizes for both within-group and between-group statistical tests are reported as Cohen’s d-values (Cohen, 1988), which are interpreted as follows: 0.1 to 0.3 indicates a small effect, 0.3 to 0.5 is an intermediate effect, and 0.5 or greater is a strong effect. A significant threshold of p < 0.05 was used for all statistical tests.

**Table 1.** Description of subjects.

	HCs	pwMS sub-groups		Group differences (p-values)		
	HCs	pwMS(BZDn)	pwMS(BZDp)	HCs vs pwMS(BZDn)	HCs vs pwMS(BZDp)	pwMS(BZDn) vs pwMS(BZDp)
N	44	61	21	-	-	-
Sex (M/F)	18/26	20/41	2/19	0.51	**0.02**	0.07
Age (years)	47 ± 12	48 ± 10	47 ± 7	0.55	0.92	0.69
Education (years)	15 ± 2	14 ± 2	13 ± 2	0.05	**0.007**	0.30
Disease duration(years)	-	17 ± 10	14 ± 6	-	-	0.19
EDSS median [IQR]	-	2.5 [2-4]	3[2.5-4]	-	-	0.12
Cognitive assessment						
SDMT	53 ±7	49 ± 12	46 ± 7	0.06	**0.004**	0.34
VGLT	65 ±7	64 ± 10	63 ± 10	0.43	0.31	0.74
COWAT	14 ± 4	13 ± 4	14 ± 4	**0.02**	0.87	0.15
BVMT-R	28 ± 5	25 ± 7	25 ±6	**0.02**	0.09	0.89

We report the mean values and standard deviations for different clinical parameters. For EDSS, the median and interquartile range (IQR) are shown.

The comparisons were performed using permutation testing with N = 5000 for all parameters except for sex, for which a chi-squared test was used. Bold values denote statistical significance at the level p < 0.05.

HCs: healthy controls, pwMS(BZDp) and pwMS(BZDn): pwMS with and without benzodiazepines. The “p” in the term for patients treated with benzodiazepines refers to the treatment status, which is positive”. Conversely, “n” in the term for patients not treated with benzodiazepines refers to the treatment status, which is “negative”.

## 3 Results

### 3.1 Behavioral data

**Fig. 2.**
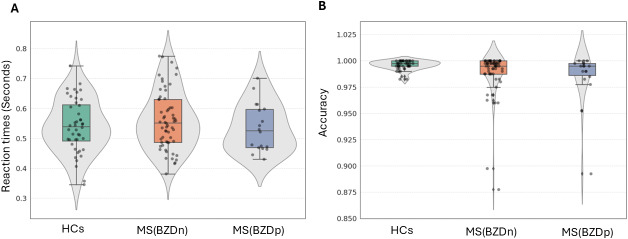
The distribution of the (A) reaction times and (B) accuracy for three groups (HCs, pwMS(BZDn), pwMS(BZDp)) are shown respectively. Each data point represents an individual participant. The Mann-Whitney tests were used to compare the reaction times and accuracy between different groups.

The distribution of reaction time and accuracy for different groups are shown in Figure 2. The group comparisons revealed a significant difference in accuracy between healthy controls (HCs) and pwMS(BZDp) patients, with HCs showing better performance (p = 0.007). No significant differences were observed in either accuracy or reaction times for any other between-group comparisons.

### 3.2 The task-induced 1/f slope steepening above and beyond the ERFs

**Fig. 3.**
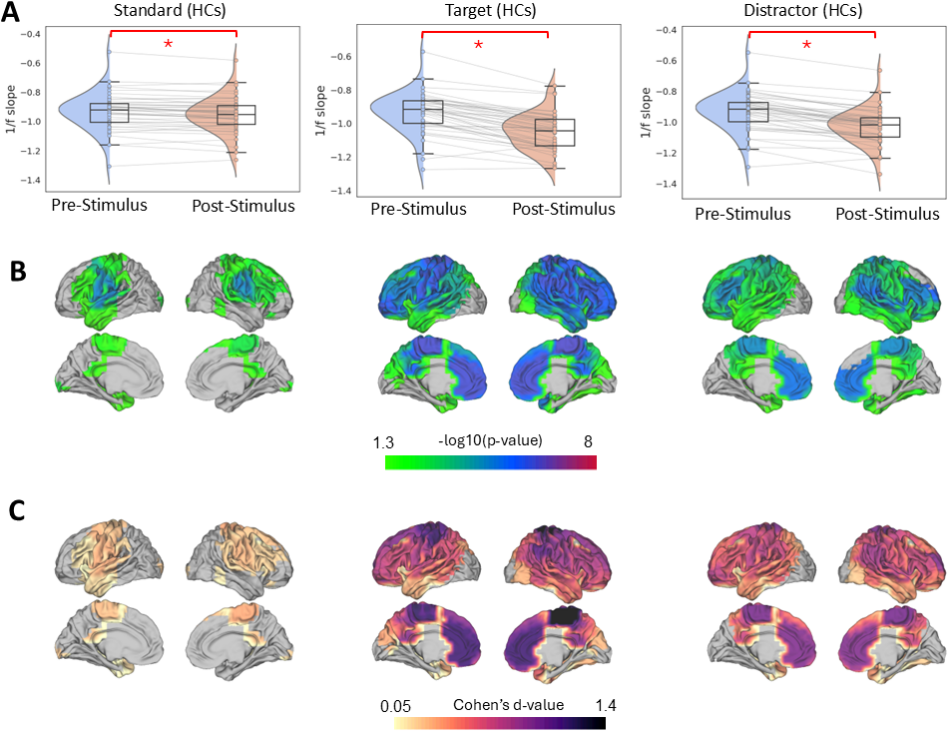
Task-induced changes in 1/f slope during the auditory oddball task in healthy controls (HCs). (A) Violin plots illustrate the distribution of whole-brain averaged 1/f slope values during pre- and post-stimulus periods across standard, target, and distractor trials. Each line represents an individual participant. Significant post-stimulus increases in 1/f slope (i.e., steepening) were observed for all conditions (Wilcoxon signed-rank test, *p < 0.0001). (B) Parcel-wise statistical maps display the spatial distribution of pre- vs. post-stimulus differences in 1/f slope across 42 brain regions. Only parcels with FDR-corrected p < 0.05 (-log_10_(p) > 1.3) are shown in color, scaled by -log_10_(p); non-significant parcels appear in light gray. (C) Effect size maps showing Cohen’s *d* for each parcel-wise comparison. The colormap spans from minimum to maximum Cohens’ d values. Only parcels with p < 0.05 (-log_10_(p) > 1.30) are shown in color; non-significant parcels appear in light gray.

**Fig. 4.**
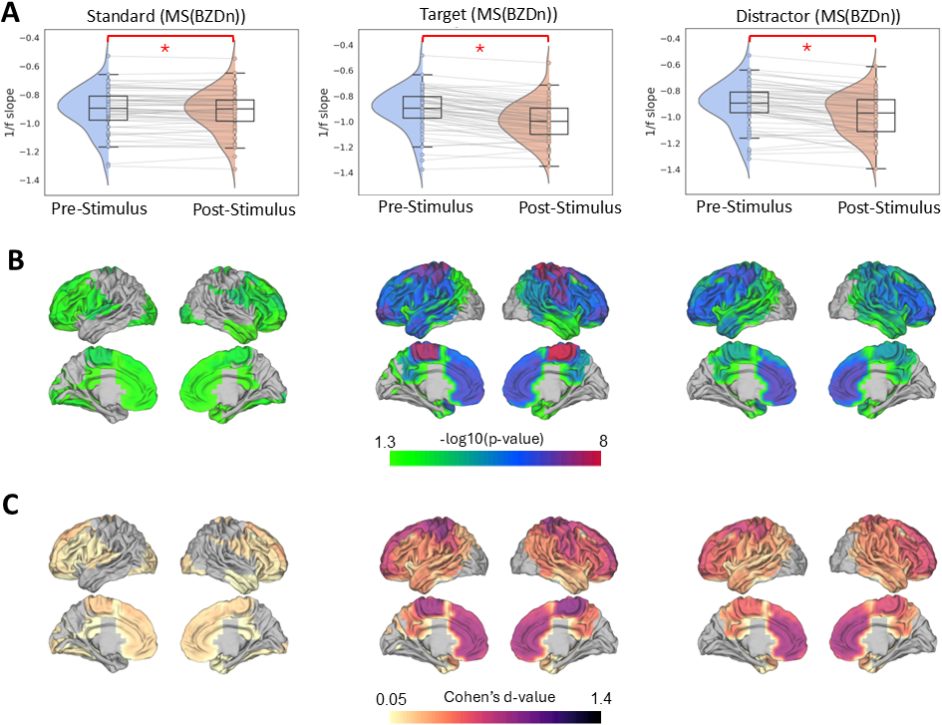
Task-induced changes in 1/f slope during the auditory oddball task in pwMS(BZDn). (A) Violin plots illustrate the distribution of whole-brain averaged 1/f slope values during pre- and post-stimulus periods across standard, target, and distractor trials. Each line represents an individual participant. Significant post-stimulus increases in 1/f slope (i.e., steepening) were observed for all conditions (Wilcoxon signed-rank test, *p < 0.0001). (B) Parcel-wise statistical maps display the spatial distribution of pre- vs. post-stimulus differences in 1/f slope across 42 brain regions. Only parcels with FDR-corrected p < 0.05 (-log_10_(p) > 1.3) are shown in color, scaled by -log_10_(p); non-significant parcels appear in light gray. (C) Effect size maps showing Cohen’s *d* for each parcel-wise comparison. The colormap spans from minimum to maximum Cohens’ d values. Only parcels with p < 0.05 (-log_10_(p) > 1.30) are shown in color; non-significant parcels appear in light gray.

Across all groups, we observed a robust and statistically significant steeper 1/f slope (indicating more inhibition) following stimulus onset for each stimulus type (standard, target, and distractor) compared to the pre-stimulus baseline. The Wilcoxon Signed-Rank Test results confirmed this effect within each group. As shown in Figure 3 for healthy controls, the 1/f slope steepening was significant for standard (W = 189, Z= -3.5, p < 0.0001, d = 0.53), distractor (W = 12, Z = -5.6, p < 0.0001, d = 0.84) and target stimuli (W = 18, Z = -5.56, p < 0.0001, d = 0.83). Similarly, in pwMS (BZDn), significant effects were observed for standard (W = 542, Z = -2.89, p = 0.003, d = 0.37), distractor (W = 91, Z = -6.13, p < 0.0001, d = 0.78), and target stimuli (W = 70, Z = -6.28 p < 0.0001, d = 0.80), as it is shown in Figure 4. In pwMS (BZDp), the 1/f slope steepening was also significant for standard (W = 55, Z = -2.10, p = 0.03, d = 0.36), distractor (W = 38, Z = -2.69, p = 0.005, d = 0.58) and target stimuli (W = 4, Z= -3.87, p < 0.0001, d = 0.84), see Figure S4.

For the parcel-level analysis, we first compared pre- and post-stimulus 1/f slopes within each cortical parcel. We then applied FDR correction across all parcels to control for multiple comparisons. Parcels showing significant differences were visualized by plotting their corrected p-values and Cohen’s d effect sizes. The resulting spatial maps for healthy controls and the two MS subgroups (MS(BZDn) and MS (BZDp)) are presented in Figures 3, 4, and S4, respectively.

In standard trials, significant steepening of the 1/f slope was predominantly observed in parietal and prefrontal parcels. Target and distractor trials, however, engaged a more extensive cortical network: in addition to parietal and prefrontal areas, motor cortex and temporal regions, involved in auditory processing, also exhibited pronounced 1/f slope steepening following stimulus onset. Non-standard stimuli showed a more spatially widespread effect than standard stimuli, consistent with increased cortical engagement when participants processed deviant sounds.

### 3.3 The task-induced increase in aperiodic intercept

Following stimulus onset, the aperiodic offset was significantly elevated in both target and distractor trials (p < 0.001). Moreover, task-related changes in the offset were highly inversely correlated with changes in the 1/f slope across all conditions (r = -0.94 to -0.98, p < 0.0001) indicating that steeper 1/f slopes were accompanied by larger increases in the offset. Comprehensive statistics on offset modulations and their relationship to the 1/f slope are provided in the Supplementary Materials (Figs. S5 and S6).

### 3.4 The effect of group and trials on 1/f slope modulation

To further evaluate the effect of group and trial on the amount of steepening in 1/f slope following the stimulus presence, we conducted a two-way ANCOVA on the rank-transformed 1/f slope modulation (post‐stimulus minus pre‐stimulus 1/f slope) with a group (HCs, pwMS(BZDn), pwMS(BZDp)) as the between-subject factor and trial type (standard, target, distractor) as the within-subject factor and age as a covariate.

**Fig. 5.**
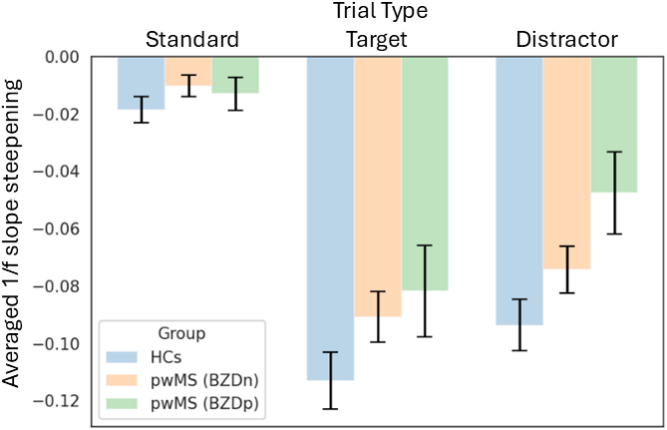
Group differences in 1/f slope modulation across trial types, based on ANCOVA results. Bar plots show the mean modulation values (averaged across all brain parcels) for each trial type and group. The three groups include healthy controls (HCs, blue), people with MS not taking benzodiazepines (pwMS(BZDn), orange), and people with MS taking benzodiazepines (pwMS(BZDp), green). The negative values indicate a task-related steepening of the 1/f slope, with larger negative modulation suggesting stronger task engagement. Error bars represent the standard error of the mean (SEM) across subjects within each group.

The main effect of age was non‐significant (F (1, 368) = 0.18, p = 0.67), and neither the age × group nor age × trial interactions were significant (p > 0.51). A main effect of group was observed, indicating that the three groups (HCs, pwMS(BZDn), pwMS(BZDp)) differed in their overall 1/f slope modulation, F(2, 368) = 6.80, p =0.001, η² = 0.03. A highly significant main effect of trial type was also observed, F(2, 368) = 94.10, p < 0.0001, η² = 0.34, demonstrating that the type of trial accounted for a substantial proportion of the variance in 1/f slope modulation. This large effect suggests that the cognitive or perceptual demands associated with different trial types substantially modulated the 1/f slope. Importantly, the interaction between group and trial type was not statistically significant, F(4, 369) = 0.65, p = 0.62, η² = 0.007. This non-significant interaction indicates that the pattern of trial effects on 1/f slope modulation was consistent across the different groups, see Figure 5.

To further examine whether the effect of age varied across brain regions, we repeated the two-way ANCOVA including age as a covariate separately for each of the 42 brain parcels. The resulting FDR-corrected p-values for the main effect of age were not significant. These results indicate that age does not significantly influence 1/f slope modulation either at the global or regional level.

#### 3.4.1 Post-hoc analysis: Between trial comparison

**Fig. 6.**
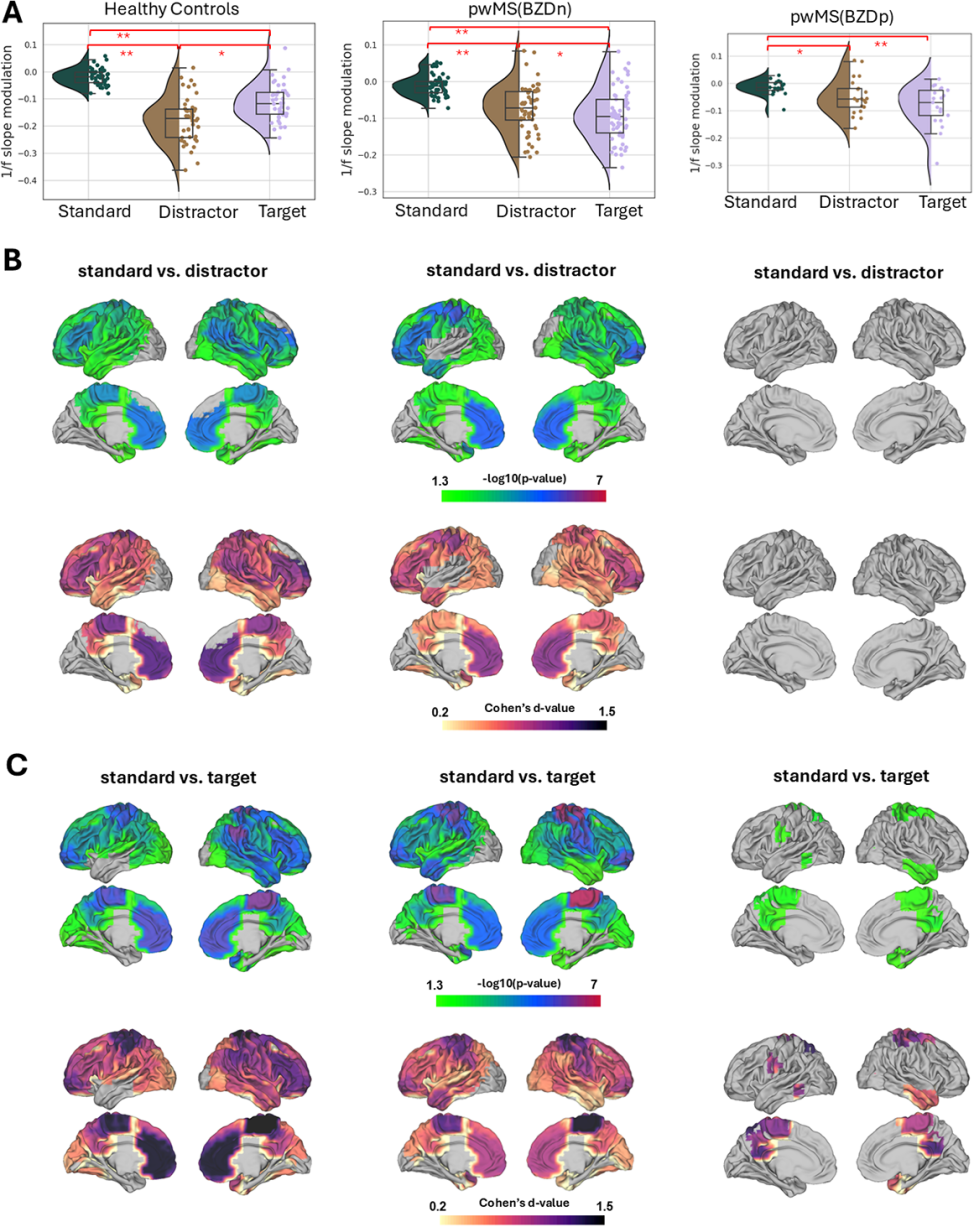
Within-group comparisons of 1/f slope modulation across trial types and spatial patterns in healthy controls and MS subgroups. (A) Violin plots show the distribution of whole-brain averaged 1/f slope modulation (post- minus pre-stimulus slope values) for standard, distractor, and target trials in each group: healthy controls (HCs), pwMS(BZDn), and (pwMS(BZDp). Each dot represents one participant. Within-group comparisons were performed using the Wilcoxon signed-rank test. Statistically significant differences are marked with asterisks (*p < 0.05, **p < 0.001). (B–C) Cortical surface maps show parcel-wise comparisons (42 brain parcels) of 1/f slope modulation between trials: (B) Standard vs. Distractor trials, (C) Standard vs. Target trials. For each comparison: Top row of each panel shows statistical significance as -log_10_(p), thresholded at p < 0.05 (-log_10_(p) > 1.3) and corrected for multiple comparisons using the FDR method. Colored regions reflect significant parcels; non-significant parcels appear in gray. Bottom row of each panel displays corresponding Cohen’s d effect sizes across the cortex.

When comparing the modulation of the 1/f slope between standard and non-standard stimuli, we observed a significantly steeper 1/f slope in response to non-standard stimuli (p < 0.001), supporting the hypothesis that increased attentional demands led to larger slope steepening, as shown in Figure 6.

In healthy controls, the 1/f slope steepening was significantly lower for standard vs. distractor (W = 13, Z = -3.57, p < 0.001, d = 0.53) and standard vs. target (W = 9, Z = -5.63,, p < 0.001, d = 0.84). A similar pattern was observed in pwMS(BZDn), with significant differences between standard vs. distractor (W = 100, Z = -6.07, p < 0.001, d = 0.77) and standard vs. target (W = 68, Z = -6.30, p < 0.001, d = 0.80). In pwMS(BZDp), we similarly found a significant difference between standard vs. distractor (W = 56, Z = 1.40, p = 0.03, d = 0.30) and standard vs. target (W = 9, Z = -3.70, p < 0.001, d = 0.80).

When comparing standard trials with target and distractor trials, parcel-wise analysis showed that 1/f slope steepening in the motor cortex was slightly higher during target trials than during distractor trials, potentially reflecting additional motor processes required by the button press response.

Interestingly, HCs showed a larger 1/f slope steepening (indicating more inhibition) in response to distractor trials compared to target trials (W = 270, Z = –5.56, p = 0.008, d = 0.83). This effect reversed direction in pwMS(BZDn) (W = 596, Z = –2.51, p = 0.01, d = 0.32), and no effect was observed in pwMS(BZDp) (W = 59, Z = –1.96, p = 0.05, d = 0.42). The parcel-wise comparisons between target and distractor stimuli did not reveal significant differences in any of the groups.

#### 3.4.2 Post-hoc analysis: Between group comparison

**Fig. 7.**
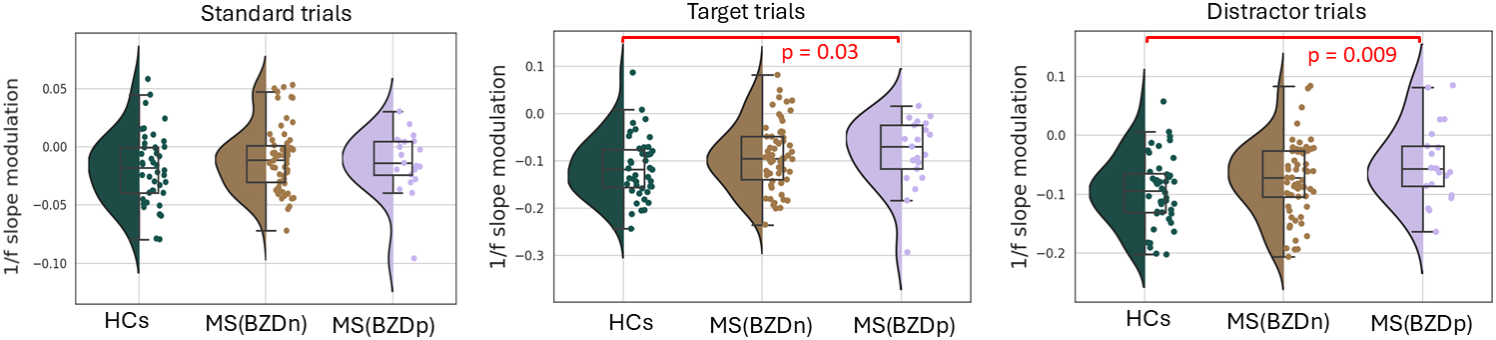
Group differences in task-induced 1/f slope modulation across trial types. Violin plots show the distribution of whole-brain averaged 1/f slope modulation values for standard, target, and distractor trials across three groups: healthy controls (HCs), pwMS(BZDn), and pwMS(BZDp). Each dot represents an individual participant. Between-group comparisons were conducted using the Mann-Whitney U test. Significant differences are indicated with red brackets and corresponding p-values.

The distribution of 1/f slope modulation averaged over the whole brain across groups for each trial types is shown in Figure 7.

During standard trials, comparisons between healthy controls (HCs) and pwMS groups did not reach statistical significance (HCs vs. pwMS(BZDn): U = 1152, Z = –1.23, p = 0.21, d = 0.24; HCs vs. pwMS(BZDp): U = 384, Z = –1.08, p = 0.27, d = 0.27). Additionally, no significant differences were observed between the two MS groups (U = 608, Z = –0.33, p = 0.73, d = 0.07).

In contrast, during target and distractor trials, the HCs showed significantly higher 1/f slope steepening compared to pwMS(BZDp): target trials (U = 308, Z = –2.15, p = 0.03, d = 0.55) and distractor trials (U = 277, Z = –2.58, p = 0.009, d = 0.68). However, comparisons between HCs and pwMS(BZDn) were not significant in either target (U = 1092, Z = –1.62, p = 0.10, d = 0.32) or distractor trials (U = 1092, Z = –1.62, p = 0.10, d = 0.32). Additionally, no significant differences were observed between the two MS groups in either target (U = 551, Z = –0.94, p = 0.34, d = 0.21) or distractor trials (U = 512, Z = –1.35, p = 0.17, d = 0.30).

Overall, these results partially support the hypothesis that healthy subjects demonstrate a more pronounced inhibitory response compared to pwMS, even though effects did not survive parcel-wise correction.

### 3.5 The correlation between the 1/f slope modulation and cognitive scores

In general, a steeper slope following the stimulus onset (indicating more inhibition) was associated with better cognitive performance. For pwMS not receiving benzodiazepines, 1/f slope modulation was significantly correlated with BVMT-R scores in both standard (r = -0.31, p = 0.01) and distractor trials (r = -0.37, p = 0.003), and also showed a negative correlation with VGLT scores in standard trials (r = -0.27, p = 0.03). In pwMS receiving benzodiazepines, a significant correlation was found between 1/f slope modulation and BVMT-R scores in distractor trials (r = -0.53, p = 0.02). No significant correlations were observed in healthy controls. Detailed results are provided in Table S2, and corresponding scatter plots are shown in Figure S7.

### 3.6 The correlation between the 1/f slope modulation following an auditory and n-back stimuli

To evaluate whether 1/f slope modulation reflects a consistent, paradigm-independent mechanism, we conducted additional analyses on 101 participants who performed both auditory oddball and the n-back working memory tasks (HCs = 34, pwMS(BZDn) = 50, pwMS(BZDp) = 17). We focused on target and distractor trials in the oddball task to align with the two-condition design (target vs. distractor) in the n-back data. We corrected the correlation analyses for the multiple comparisons.

**Fig. 8.**
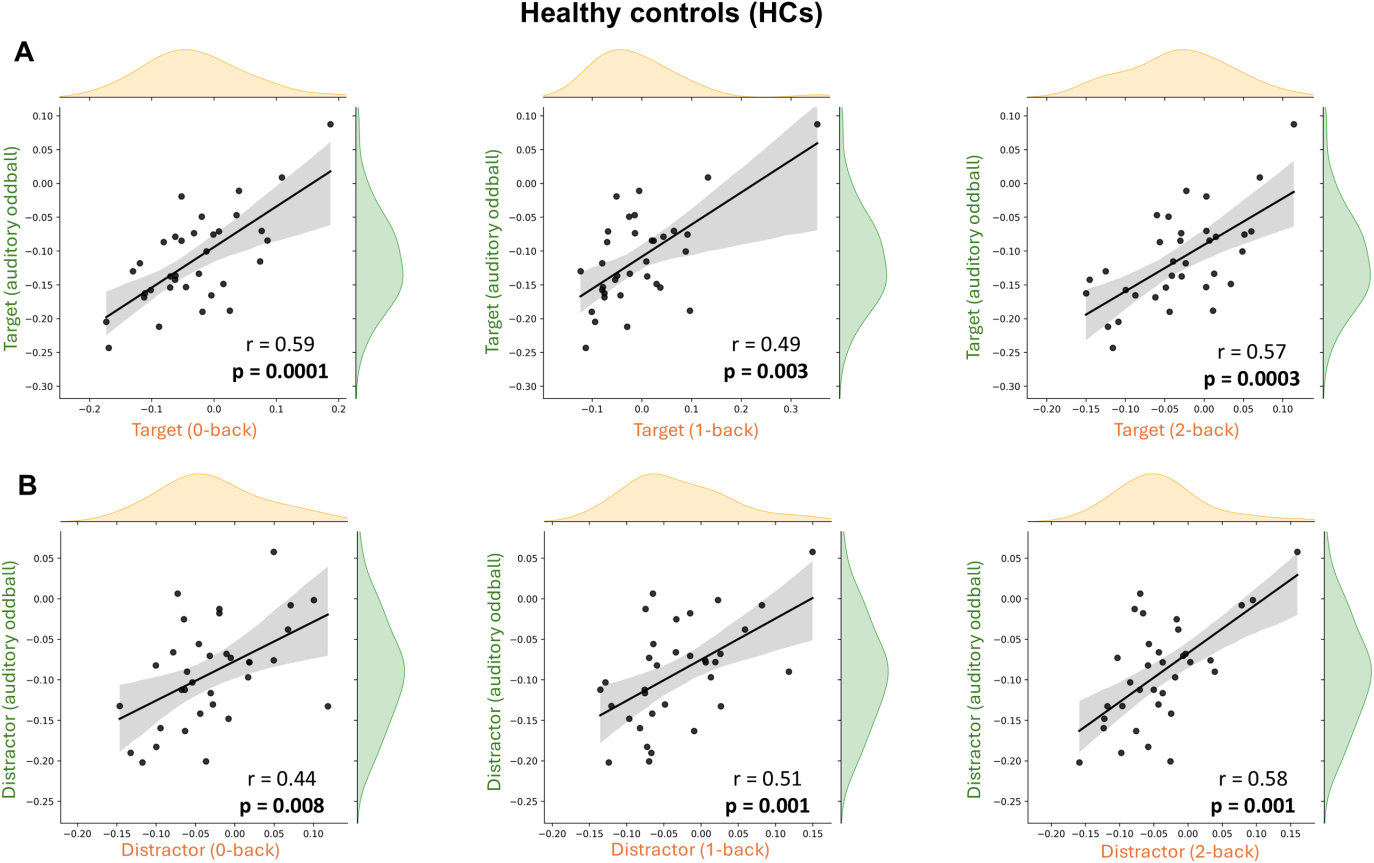
Cross-paradigm correlation of 1/f slope modulation between the auditory oddball and n-back tasks in healthy controls (HCs). Scatter plots display the relationship between 1/f slope modulation in the auditory oddball task (y-axis) and the n-back task (x-axis) across three working memory load conditions: 0-back, 1-back, and 2-back. (A) Correlations for target trials across the two tasks. (B) Correlations for distractor trials across the two tasks. Each dot represents one participant; shaded bands indicate 95% confidence intervals for the regression line. All Spearman’s correlation coefficients (*r*) and corresponding p-values are reported in each plot.

**Fig. 9.**
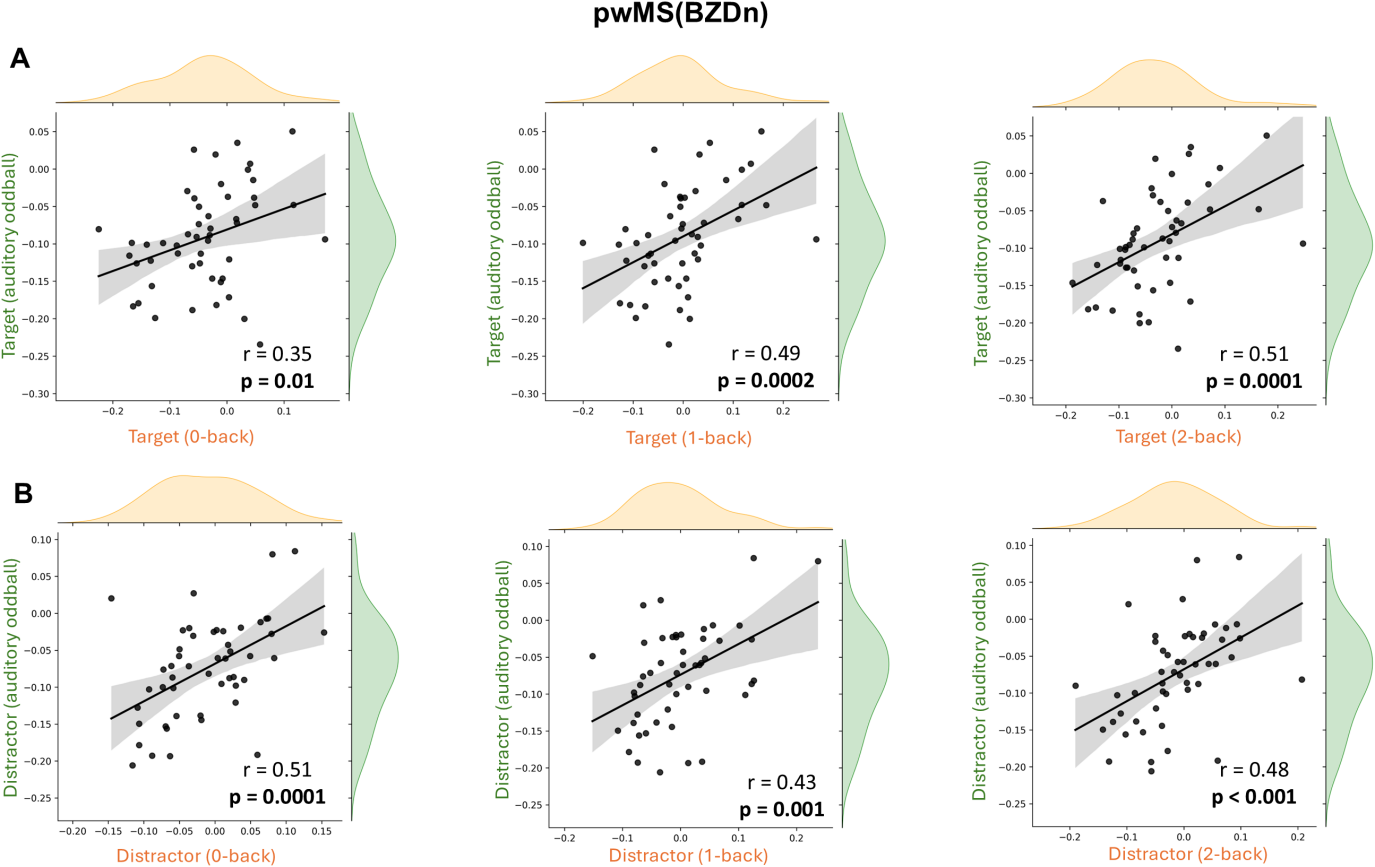
Cross-paradigm correlation of 1/f slope modulation between the auditory oddball and n-back tasks in pwMS(BZDn). Scatter plots display the relationship between 1/f slope modulation in the auditory oddball task (y-axis) and the n-back task (x-axis) across three working memory load conditions: 0-back, 1-back, and 2-back. (A) Correlations for target trials across the two tasks. (B) Correlations for distractor trials across the two tasks. Each dot represents one participant; shaded bands indicate 95% confidence intervals for the regression line. All Spearman’s correlation coefficients (*r*) and corresponding p-values are reported in each plot.

Healthy controls and pwMS(BZDn) showed a significant positive correlation in 1/f slope modulation between the two tasks across all n-back load levels (0-back, 1-back, 2-back), as shown in Figures 8 and 9, respectively. Individuals who showed steeper (or flatter) 1/f slope following stimulus onset in the oddball task tended to demonstrate a similar pattern in the n-back task. Among pwMS(BZDp), correlations did not reach significance for the 0-back and 1-back target and 0-back distractor conditions but exhibited a similar trend, see Figure S8.

## 4 Discussion

This study explores the neurophysiological underpinnings of cognitive impairments in multiple sclerosis through the lens of the aperiodic 1/f slope modulation by leveraging MEG data during auditory oddball and visual-verbal n-back tasks. We investigated the modulation of the 1/f slope as an indicator of excitation/inhibition (E/I) balance and its potential role as a paradigm-independent marker of cognitive control. Our findings revealed several key insights into the neural mechanisms of cognitive dysfunction in MS and underscore the potential utility of aperiodic spectral components in cognitive research.

### 4.1 The 1/f slope steepening beyond event-related fields

Across all groups and stimulus conditions, we observed a significant post-stimulus increase in the steepness of the 1/f slope, above and beyond any changes explained by conventional event-related fields. Simultaneously, we observed a robust increase in the aperiodic intercept, indicating that this inhibitory shift is accompanied by a global up-shift in broadband cortical activity. This finding is consistent with prior works showing that sensory input triggers an immediate global shift toward steepening in 1/f spectral slope and increased inhibition in cortical circuits (Akbarian et al., 2024; Gyurkovics et al., 2022; Kałamała et al., 2023). Non-standard stimuli induced larger 1/f slope steepening, suggesting that higher cognitive demands lead to larger shifts in the slope steepening (Gyurkovics et al., 2022; Kałamała et al., 2023; Lu et al., 2024). However, some studies have reported the opposite effect (i.e., flattening of the slope) in response to the higher cognitive demanding tasks and salience conditions. Arnett et al. (2022) showed that in a visual dual-task paradigm with typically developing children, higher working‐memory, and cognitive demands led to a flatter aperiodic slope (over 1–50 Hz), a pattern interpreted as heightened local network recruitment; notably, they also observed transient slope steepening immediately prior to correct target detections, suggesting that global network coordination enhances attentional efficiency. More recently, Mocchi et al. (2024) demonstrated that salient tones in an auditory oddball task induced a sustained flattening of the aperiodic slope (over 20–45 Hz), an effect that persisted into the very next trial. While, similar to our findings, Monchy et al. (2024) reported a steeper post-stimulus slope, the aperiodic slope remained flatter during the task, both before and after stimulus onset, compared to resting state. Together, these results suggest that 1/f dynamics index distinct modes of cortical engagement rather than serving as a unidirectional signature of cognitive control.

#### 4.1.1 Spatial distribution of 1/f slope steepening

When examining FDR-corrected, parcel‐level aperiodic 1/f slope changes in healthy controls and pwMS not on benzodiazepines, we found that frequent (standard) stimuli produced a localized steepening of the 1/f slope primarily within parietal and prefrontal cortices. In contrast, infrequent or task-salient stimuli (targets and distractors) drove a far more extensive topography of slope steepening: alongside parietal and prefrontal parcels, significant increases appeared in temporal areas and sensorimotor cortex. This pattern may reflect a global inhibitory reset, triggered by a sensory input, that drives broad 1/f slope steepening. This could reflect a rapid neural inhibition of on-going activity to facilitate transmission of stimulus information (Munakata et al., 2011; Polich, 2007). For standard stimuli, predictable sounds, parietal–prefrontal gating might be sufficient to process the input. However, when sounds carry behavioral relevance, either as targets requiring a response or as distractors that must be suppressed, additional recruitment of auditory sensory and frontoparietal control circuits sharpens discrimination and guides orienting responses (Gyurkovics et al., 2022; Polich, 2007). Thus, the additional involvement of temporal-parietal cortex during non‐standard trials could indicate its critical role in detecting and encoding auditory deviations (Howard et al., 2000; Nourski, 2017), while the more widespread effect on frontal and parietal areas likely reflects the engagement of top-down attentional and executive processes necessary for perceptual discrimination and goal-directed behavior.

#### 4.1.2 Enhanced 1/f slope steepening in motor cortex during overt responses

Beyond sensory and attentional circuits, we also observed larger steepening of the 1/f slope in motor cortex on trials requiring button-press responses. This suggests a response-specific stage of inhibitory control: once a decision to respond is reached, motor areas undergo a transient boost in inhibitory drive that gates motor output until the precise moment of execution (Bestmann & Duque, 2016). This effect reflects two complementary inhibitory processes (Duque et al., 2010, 2012): One that resolves competition by suppressing representations of incorrect responses to ensure the appropriate response is selected, and another that implements impulse control by inhibiting the selected response itself to prevent its premature execution. The transient increase in 1/f steepness likely reflects the activity of local GABAergic interneurons suppressing spontaneous motor cortex firing, permitting a rapid and precisely timed release of inhibition at response onset. This motor-related steepening in 1/f slope could also reflect response-locked event-related fields (ERFs), which were not explicitly controlled for in our analysis since our baseline correction accounted only for stimulus-locked ERFs.

### 4.2 Phasic inhibitory control deficits in MS revealed by 1/f slope modulation

Similar to our previous findings (Akbarian et al., 2024), people with MS treated with benzodiazepines showed reduced 1/f slope modulation in response to distractor stimuli compared to HCs. This suggests that benzodiazepine use may disrupt inhibitory neural mechanism (Nicholson et al., 2018), potentially contributing to the cognitive deficits reported in the literature on benzodiazepines (Barker et al., 2004; Zetsen et al., 2022). Moreover, while healthy controls showed a steeper 1/f slope following distractor versus target trials, potentially reflecting up-regulation of GABAergic inhibition under increased conflict, pwMS exhibited the reverse pattern (flatter slope after distractors than targets). The pre-stimulus (baseline) 1/f slopes did not differ between groups, indicating similar tonic inhibition. Thus, the observed crossover cannot arise from a baseline shift or uniform scaling but instead could reflect a deficit in dynamic-range recruitment: while pwMS can achieve normal baseline inhibition to well-practiced targets, they seem to lack the additional capacity to further boost inhibition when distractors demand extra suppression. This selective impairment of phasic inhibitory control points to an excitation/inhibition imbalance in pwMS that manifests only under high-demand conditions. This finding resonates with prior evidence of synaptic loss and dysregulation of inhibitory control in MS (Huiskamp et al., 2022; Zoupi et al., 2021) and highlights the potential of aperiodic spectral slope in capturing subtle neurophysiological alterations that may underlie cognitive deficits. Importantly, directional changes in the 1/f spectral slope (steepening or flattening) offer a direct and interpretable marker of shifts in E/I balance, in contrast to the often challenging interpretation of ERFs polarity (Hajizadeh et al., 2019).

### 4.3 Association between 1/f slope dynamics and cognitive scores

Also, in line with our previous work (Akbarian et al., 2024), we observed that the task-induced 1/f slope steepening exhibited a significant negative correlation with offline visuospatial memory, as measured by the BVMT-R, in pwMS(BZDn). Specifically, a steeper slope following the stimulus onset (indicating more inhibition) was associated with better performance, suggesting that enhanced inhibitory neural processes may support cognitive function in this domain. Recent multimodal neuroimaging studies have reported positive correlations between hippocampal GABA concentrations, receptor density, and both visuospatial and verbal memory in pwMS (Huiskamp et al., 2023; Zhang et al., 2024), further emphasizing the role of inhibitory neurotransmission in cognitive performance. Moreover, visuospatial memory has emerged as a critical cognitive domain for distinguishing various cognitive profiles among pwMS (Van Dam et al., 2024), underscoring its clinical relevance. Future research should explore this relationship in greater detail to elucidate the underlying mechanisms, thereby advancing the potential of 1/f slope modulation as a clinical biomarker for cognitive impairment in pwMS.

Several factors may explain the lack of association between 1/f slope modulation and neuropsychological test performance across other groups and cognitive tests. First, the auditory oddball task may impose a relatively modest cognitive load, limiting its sensitivity to detect individual differences in higher-order cognitive functions. Also, it is possible that the tsk-induced 1/f slope modulation captures a distinct aspect of neural function that is not directly reflected in the selected neuropsychological measures. Finally, it is possible that our group-level sample sizes were insufficient to detect a reliable relationship between cognitive performance and slope modulation, particularly given that slope modulation was calculated as a difference score (post- minus pre-exponent), and such difference measures are known to have weaker psychometric properties compared to absolute measures (Hedge et al., 2018; Von Bastian et al., 2020). Future research employing larger samples and a wider array of cognitive tasks varying in complexity may offer a more comprehensive assessment of these relationships.

### 4.4 Cross-paradigm consistency of 1/f slope modulation

A salient finding of our study is the significant positive correlation in 1/f slope modulation between the auditory oddball and n-back tasks among HCs and pwMS(BZDn). Subjects who show larger (or smaller) 1/f slope changes in one task tend to show a similar pattern in the other. This cross-task consistency supports that the 1/f slope functions as a trait-like indicator of cognitive control, reflecting underlying E/I balance or top-down regulatory mechanisms engaged across diverse cognitive demands. Recent work using different approaches (multivariate pattern analysis) across three tasks has similarly demonstrated cross-task generalization of 1/f aperiodic components (Lu et al., 2024), further bolstering its role as a paradigm-independent neurophysiological marker. In contrast to the overall pattern observed, the subgroup of pwMS receiving benzodiazepine treatment (pwMS(BZDp)) did not show significant cross-task correlations. The lack of significant findings might be due to the relatively small sample size in the pwMS(BZDp) subgroup, which might have limited the statistical power needed to detect subtle effects.

### 4.5 Limitations and future works

While our study provides novel insights, some limitations must be acknowledged. One potential limitation of our study is the restricted time window around stimulus onset. Due to the constraints of our task paradigm, we confined the analysis to a 500-ms window, which reduced the frequency resolution to 2 Hz. Despite this, power spectra were modeled with high model fit during aperiodic parametrization in all our analyses (See Supplementary Materials, Fig. S9). Future studies may benefit from extending the time window to improve frequency resolution, with the caveat that this would lead to decreased temporal resolution which may pose a problem if aperiodic changes are relatively short-lived.

It is also important to note that while the 1/f slope is often used as an indirect measure of excitatory/inhibitory balance, its biophysical origins remain debated. Baranauskas et al. (2012) demonstrated that the 1/f scaling is a result of the rate fluctuations in the cortical up and down states transition in the brain, but this study has been done using data recorded under general anesthesia in rodents and may not fully generalize to human cortex. Muthukumaraswamy and Liley (2018) provide a complementary perspective, attributing the 1/f power-law scaling to the damping properties of harmonic oscillators. However, a growing body of human EEG/MEG research supports the view that altered aperiodic slopes index E/I disruptions in clinical and age‐related contexts. For instance, patients with schizophrenia exhibit a significant flatter slope at rest consistent with cortical disinhibition (Peterson et al., 2023), and children with ADHD show reduced slope steepness during task engagement suggestive of impaired phasic inhibition (Dakwar-Kawar et al., 2024; Robertson et al., 2019). Moreover, healthy aging is accompanied by a gradual flattening of the 1/f slope, reflecting a shift in excitation/inhibition balance across the lifespan (Voytek et al., 2015). Importantly, Waschke et al. (2021) showed that pharmacological manipulation of E/I balance with anesthetics shifts the 1/f slope: propofol, which enhances inhibition, makes it steeper, whereas ketamine, which boosts excitation, flattens it, consistent with their known neurochemical profiles. Together, these human studies in awake participants reinforce the translational validity of 1/f slope modulation as a potential marker of E/I balance and further support our interpretation of altered inhibitory dynamics in pwMS.

Building on our findings, future research should explore several avenues to deepen our understanding of E/I balance disruptions in pwMS. Longitudinal studies which track changes in 1/f slope modulation over time could elucidate the progression of these disruptions and their relationship with cognitive decline. In addition, employing a broader spectrum of cognitive paradigms with varying demands would better capture the relationship between 1/f slope modulation and specific cognitive domains. Integrating aperiodic spectral measures with structural and functional imaging biomarkers may enhance the predictive power for cognitive impairments in MS. Moreover, employing a variety of preprocessing and analytical methods can help assess the robustness of the observed effects across different methodological choices, such as source reconstruction techniques, that may impact the shape of the power spectrum. Also, we used the first principal component as a time series of each parcel, future studies can investigate the multi‐component approaches. However, because any preprocessing modifications would uniformly affect all participants, we do not expect them to alter our conclusions regarding group comparisons.

## 5 Conclusion

This study underscores the potential of the aperiodic 1/f slope as a sensitive and paradigm-independent neurophysiological marker of cognitive control and E/I balance. Attenuated distractor-related modulation among people with MS may reflect a phasic inhibition deficit that contributes to their cognitive impairment. The observed correlations across distinct cognitive tasks suggest the utility of the 1/f slope in capturing stable aspects of cognitive processing. These findings pave the way for future research using aperiodic spectral components to better understand cognitive impairments in MS and other neurological conditions.

## Supplementary Material

Supplementary Material

## Data Availability

Data and code are available at reasonable request from the corresponding author.
