# Supplementary material for: Task-induced 1/f slope modulation as a paradigm-independent marker of cognitive control in multiple sclerosis
Source: Imaging Neurosci (Camb). 2025 Oct 30;3:IMAG.a.973. doi: 10.1162/IMAG.a.973 (PMC12576845; doi:10.1162/IMAG.a.973)
Supplement: Supplementary Material [file IMAG.a.973_supp.pdf]

Supplementary Materials

Task-induced 1/f slope modulation as a paradigm-independent marker of cognitive control in multiple sclerosis

Fahimeh Akbarian[1,2*], Máté Gyurkovics[3], Marie B D'hooghe[4,5],
Miguel D'haeseleer[4,5,6], Guy Nagels[2,6,7], Jeroen Van Schependom[1,2*]

This supplementary document provides detailed supporting information for the manuscript titled *"Task-induced 1/f slope modulation as a paradigm-independent marker of cognitive control in multiple sclerosis."* It includes:

- **Variance analysis** of principal components across brain parcels (Figures S1-S3, Table S1).
- **Additional results** from the auditory oddball task, including group-specific 1/f slope changes, intercept dynamics, and spatial distributions (Figures S4-S6).
- **Exploratory correlations** between spectral measures and cognitive performance across multiple domains (Figure S7, Table S2).
- **Cross-task validation**, showing consistent 1/f slope modulation patterns across oddball and n-back tasks (Figure S8).
- **Model fitting quality** assessment for the specparam algorithm (Figure S9).
- **Parcellation atlas details** listing the 42 cortical regions used in source-level analysis (Table S3).

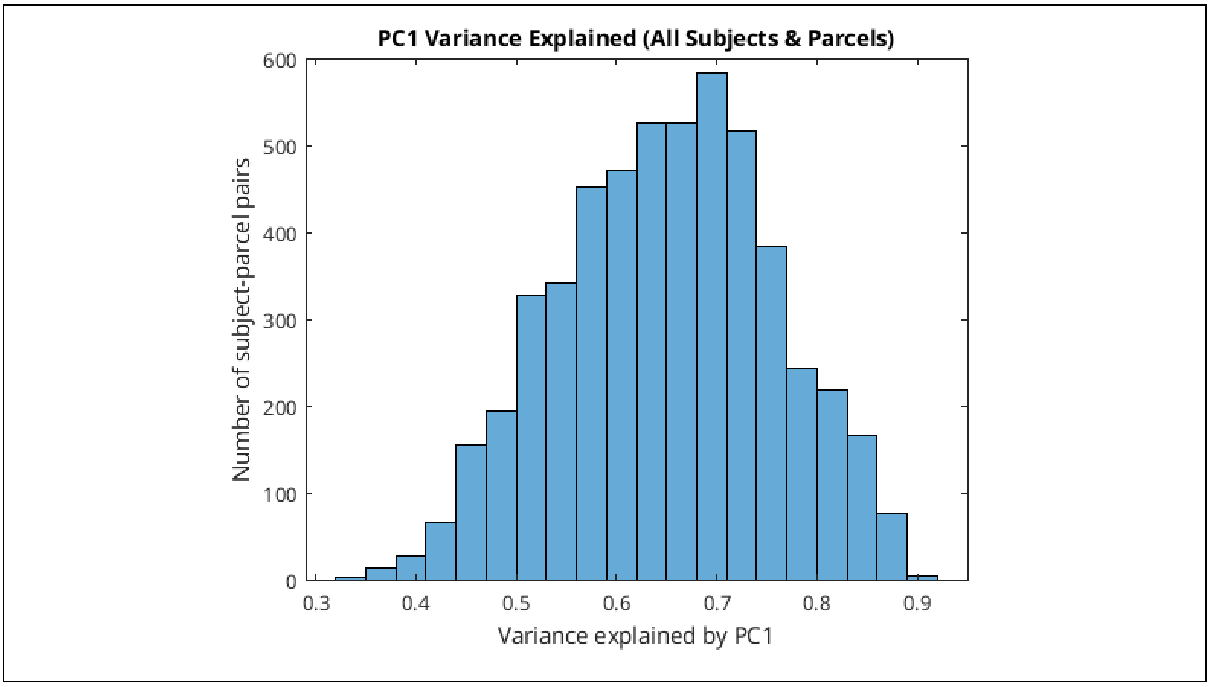

Figure S1. The distribution of variance explained by first principal component (PC1) across brain parcels and subjects**.** For each subject and brain parcel, we performed principal component analysis (PCA) and calculated the variance explained by the first principal component (PC1). This figure shows the distribution of these variance values across all subject-parcel pairs, providing an overview of how much of the signal variance can be captured by the dominant component across the dataset. Higher values indicate a greater contribution of PC1 to the overall signal within a given parcel.

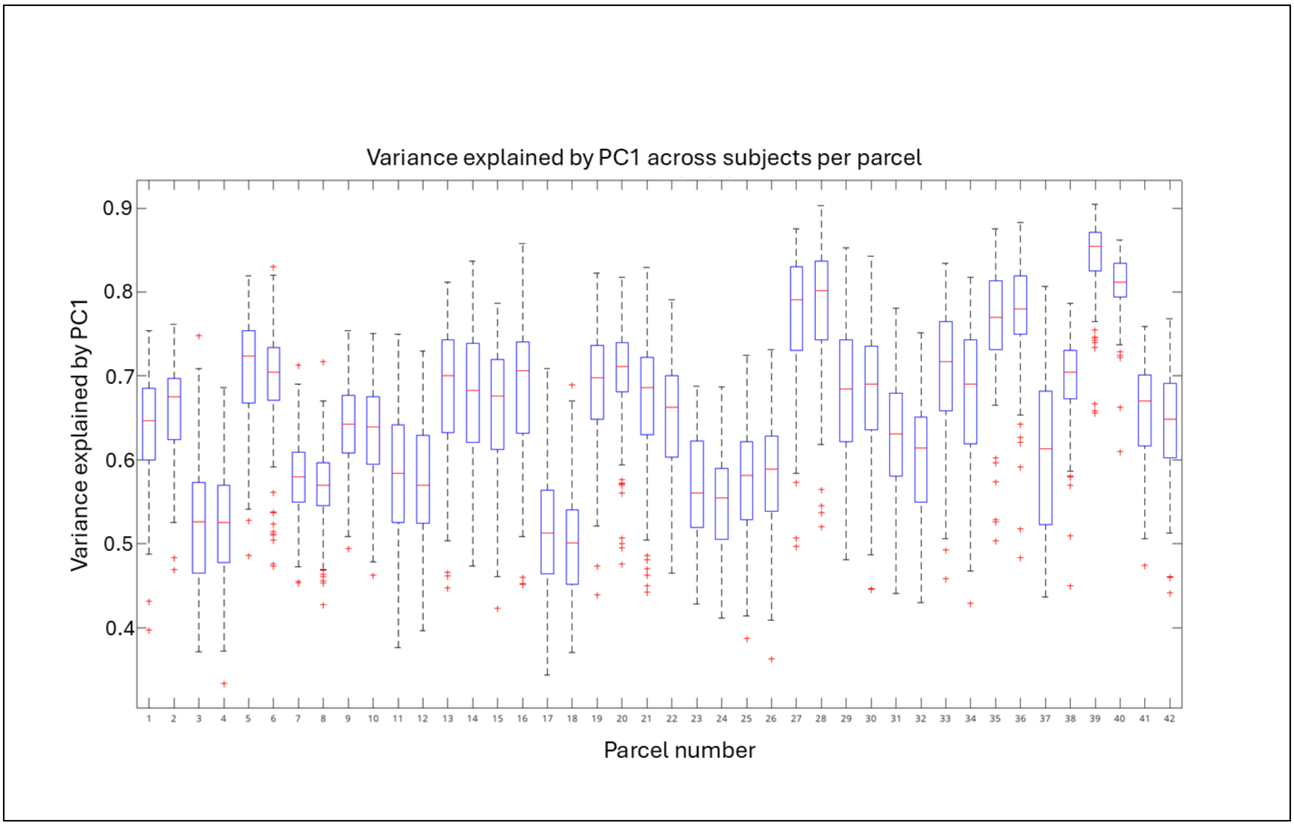

Figure S2. Boxplot of variance explained by the first principal component (PC1) for each brain parcel. This figure presents a boxplot summarizing the distribution of variance explained by PC1 across subjects for each individual brain parcel (ROI 1 to ROI 42). Each box represents the interquartile range (IQR) of PC1 variance values for a given parcel, with the median shown as a horizontal line and potential outliers displayed with red "+" symbols. These values reflect how consistently the first principal component captures signal variance across subjects within each parcel. A full list and description of all brain parcels is provided in Table S3.

Table S1. This table provides statistics visualized in Figure S2.

ROI	Mean	Standard deviation	Minimum	Maximum
1	0.64	0.06	0.40	0.75
2	0.66	0.06	0.47	0.76
3	0.53	0.07	0.37	0.75
4	0.53	0.07	0.33	0.69
5	0.71	0.06	0.49	0.82
6	0.69	0.07	0.47	0.83
7	0.58	0.05	0.45	0.71
8	0.57	0.05	0.43	0.72
9	0.64	0.05	0.49	0.75
10	0.63	0.06	0.46	0.75
11	0.58	0.08	0.38	0.75
12	0.57	0.07	0.40	0.73
13	0.68	0.08	0.45	0.81
14	0.67	0.08	0.47	0.84
15	0.66	0.08	0.42	0.79
16	0.69	0.08	0.45	0.86
17	0.51	0.07	0.34	0.71
18	0.50	0.06	0.37	0.69
19	0.69	0.07	0.44	0.82
20	0.70	0.07	0.48	0.82
21	0.67	0.08	0.44	0.83
22	0.65	0.07	0.46	0.79
23	0.57	0.06	0.43	0.69
24	0.55	0.06	0.41	0.69
25	0.57	0.07	0.39	0.72
26	0.58	0.07	0.36	0.73
27	0.77	0.08	0.50	0.88
28	0.78	0.08	0.52	0.90
29	0.68	0.09	0.48	0.85
30	0.68	0.07	0.45	0.84
31	0.63	0.07	0.44	0.78
32	0.60	0.07	0.43	0.75
33	0.70	0.08	0.46	0.83
34	0.68	0.09	0.43	0.82
35	0.76	0.07	0.50	0.88
36	0.78	0.07	0.48	0.88
37	0.61	0.09	0.44	0.81
38	0.70	0.05	0.45	0.79
39	0.84	0.05	0.66	0.91
40	0.81	0.04	0.61	0.86
41	0.66	0.06	0.47	0.76
42	0.64	0.07	0.44	0.77

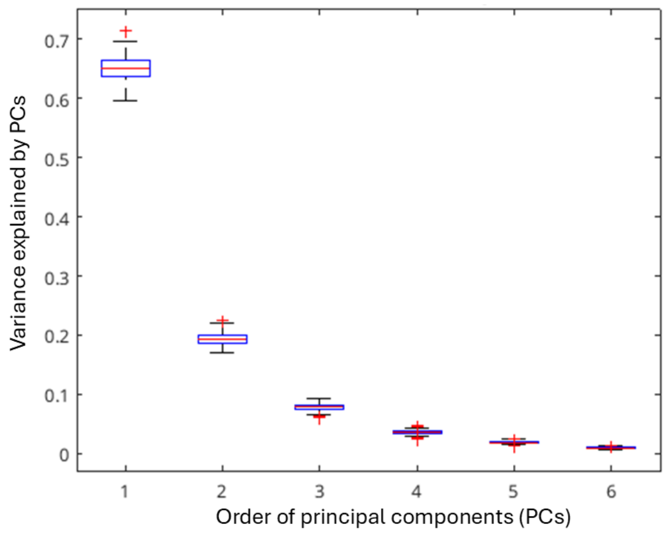

Figure S3. Variance explained by the first six principal components (PC1–PC6) across subjects. This boxplot illustrates the distribution of variance explained by each of the first six principal components, averaged across all brain parcels and subjects. For each subject and each parcel, principal component analysis (PCA) was performed individually, and the proportion of variance explained by each component was calculated. The figure summarizes these values across all subjects and parcels, showing that the first principal component (PC1) consistently explains the largest proportion of variance in the data. Subsequent components (PC2–PC6) explain progressively smaller amounts of variance. The boxplots represent the interquartile range (IQR), with the red "+" symbols indicating outliers.

Results:

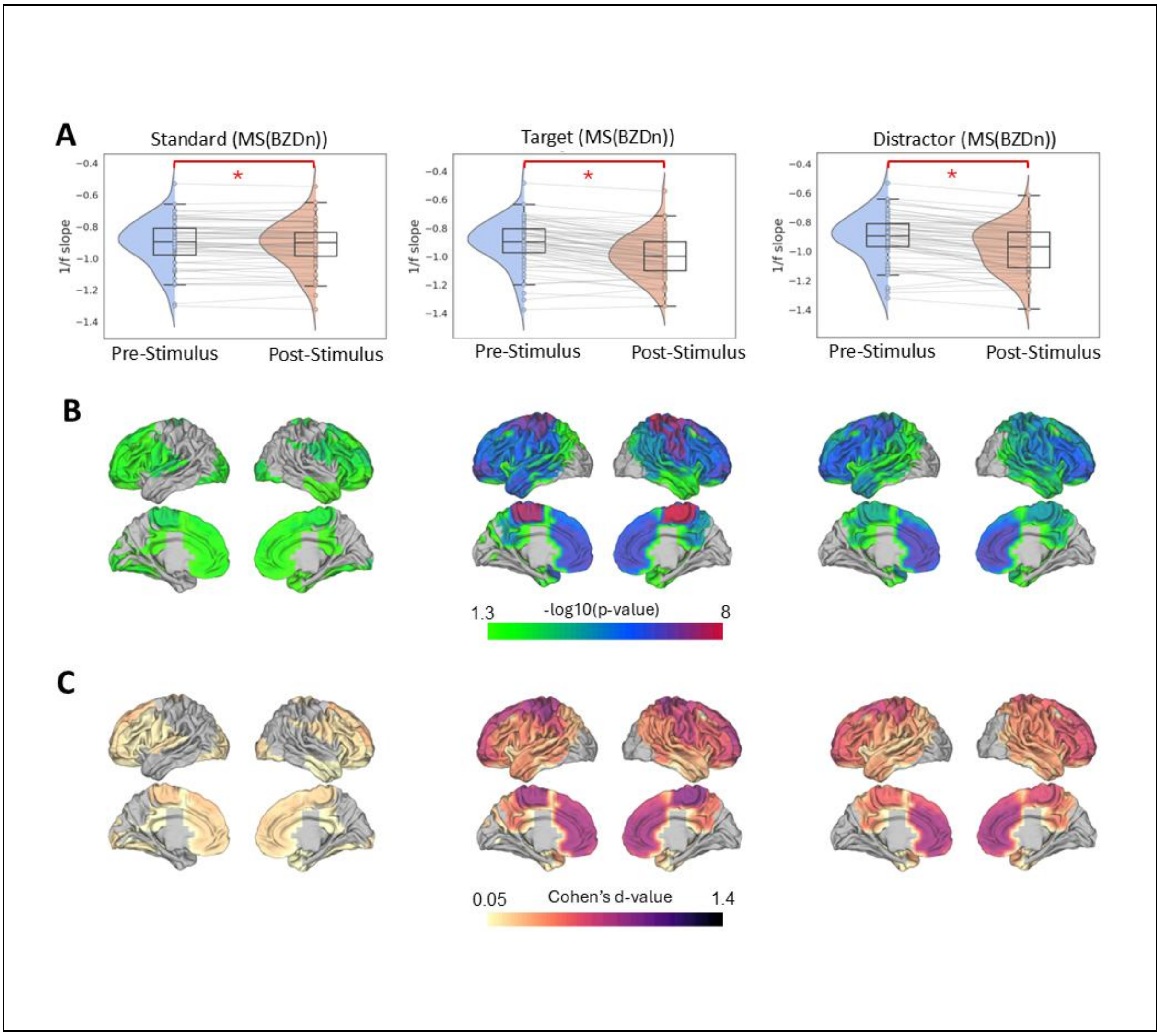

Figure S4. Task-induced changes in 1/f slope during the auditory oddball task in pwMS(BZDp). **(A)** Violin plots illustrate the distribution of whole-brain averaged 1/f slope values during pre- and post-stimulus periods across standard, target, and distractor trials. Each line represents an individual participant. Significant increase in 1/f slope (i.e., steepening) were observed for all conditions (Wilcoxon signed-rank test, *$p < 0.0001$). **(B)** Parcel-wise statistical maps display the spatial distribution of pre- vs. post-stimulus differences in 1/f slope across 42 brain regions. Only parcels with FDR-corrected $p < 0.05$ ($-\log_{10}(p) > 1.3$) are shown in color, scaled by $-\log_{10}(p)$; non-significant parcels appear in light gray. **(C)** Effect size maps showing Cohen's *d* for each parcel-wise comparison. The colormap spans from minimum to maximum Cohens' d values. Only parcels with $p < 0.05$ ($-\log_{10}(p) > 1.30$) are shown in color; non-significant parcels appear in light gray.

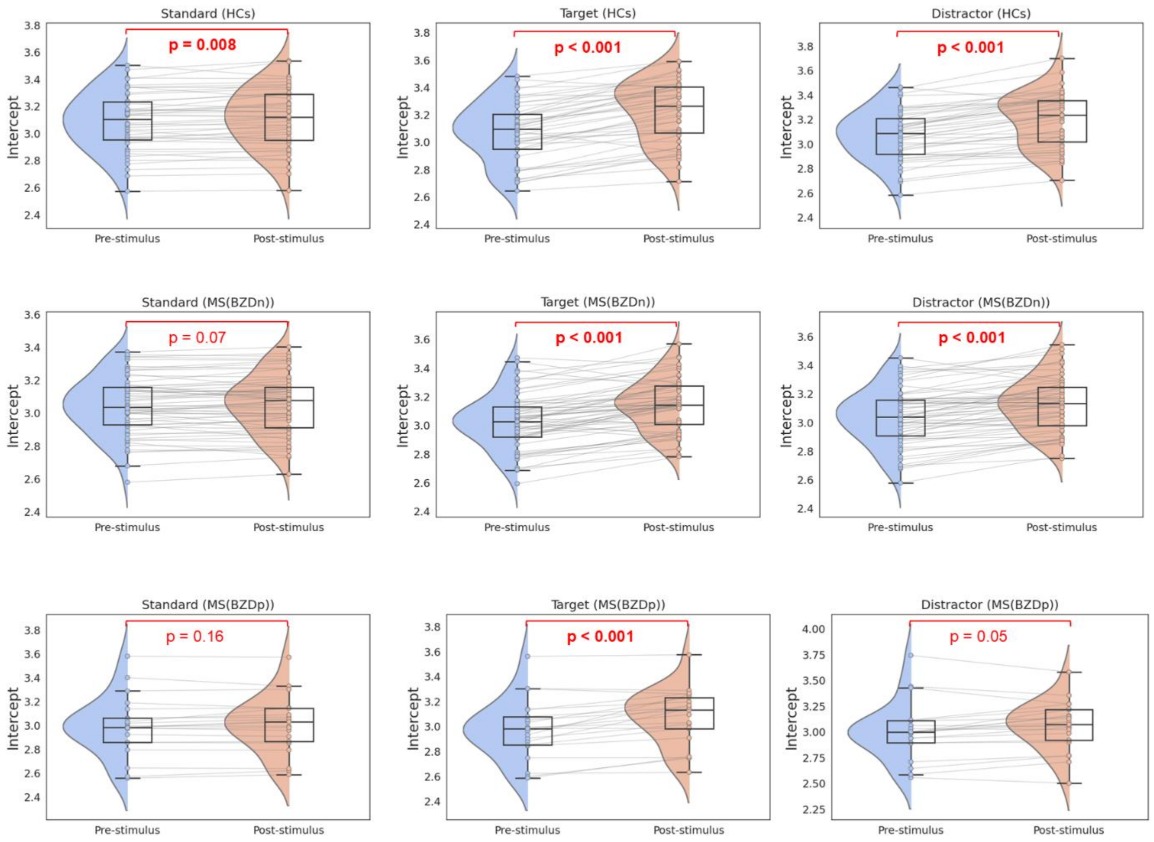

Figure S5. Task-induced changes in intercept during the auditory oddball task. Violin plots illustrate the distribution of whole-brain averaged 1/f intercept (offset) values during pre- and post-stimulus periods across standard, target, and distractor trials for HCs, pwMs(BZDn), pwMS(BZDp). Each line represents an individual participant.

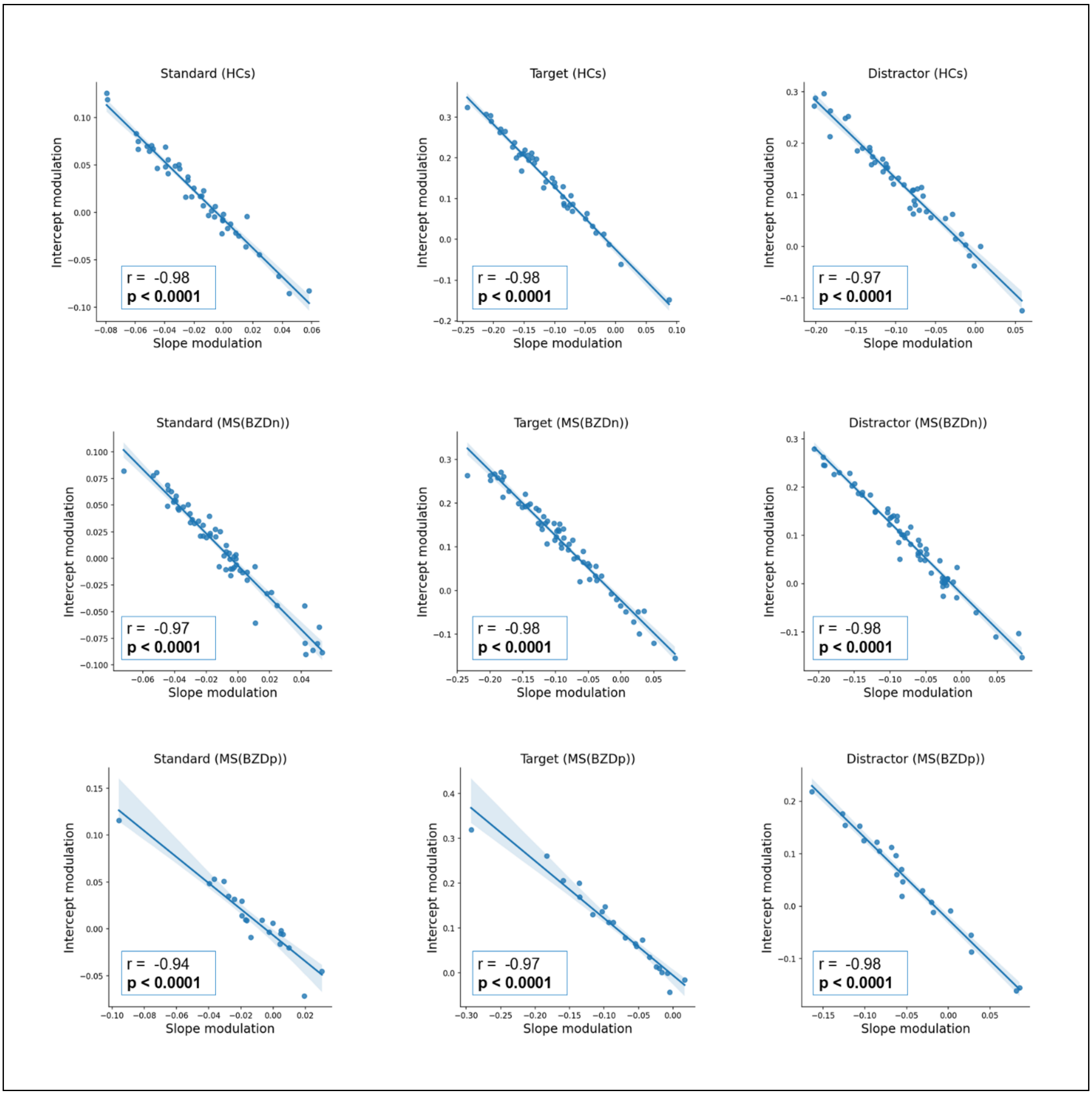

Figure S6. Correlation between task-induced changes in spectral intercept and 1/f slope across trial types and groups. This figure presents scatter plots and corresponding statistical results from correlation analyses examining the relationship between task-induced changes in the spectral intercept and 1/f slope during the auditory oddball task. Correlations are shown separately for each trial type (standard, target, and distractor) and for each group: healthy controls (HCs), people with MS not taking benzodiazepines (pwMS(BZDn)), and people with MS taking benzodiazepines (pwMS (BZDp)).
Each point represents an individual participant's average change in intercept and 1/f slope across brain parcels for a given trial type. The analysis aims to assess whether modulations in the intercept and slope components of the aperiodic activity are systematically related, potentially reflecting shared underlying mechanisms of task-related neural dynamics

	Standard trial	Target trials	Distractor trials
**BVMT-R**	HCs: r = 0.14, p = 0.35<br>**pwMS(BZDn): r = -0.31, p = 0.01**<br>pwMS(BZDp): r = 0.05, p = 0.84	HCs: r = 0.08, p = 0.58<br>pwMS(BZDn): r = -0.23, p = 0.07<br>pwMS(BZDp): r = -0.27, p = 0.27	HCs: r = 0.23, p = 0.11<br>**pwMS(BZDn): r = -0.37, p = 0.003**<br>**pwMS(BZDp): r = -0.53, p = 0.021**
**SDMT**	HCs: r = -0.15, p = 0.31<br>pwMS(BZDn): r = -0.21, p = 0.09<br>pwMS(BZDp): r = 0.21, p = 0.39	HCs: r = 0.001, p = 0.99<br>pwMS(BZDn): r = -0.04, p = 0.72<br>pwMS(BZDp): r = 0.08, p = 0.74	HCs: r = 0.01, p = 0.90<br>pwMS(BZDn): r = -0.17, p = 0.17<br>pwMS(BZDp): r = -0.03, p = 0.87
**VGLT**	HCs: r = 0.14, p = 0.36<br>**pwMS(BZDn): r = -0.27, p = 0.03**<br>pwMS(BZDp): r = -0.03, p = 0.87	HCs: r = 0.08, p = 0.56<br>pwMS(BZDn): r = -0.16, p = 0.19<br>pwMS(BZDp): r = -0.24, p = 0.33	HCs: r = 0.19, p = 0.19<br>pwMS(BZDn): r = -0.25, p = 0.05<br>pwMS(BZDp): r = -0.09, p = 0.70
**COWAT**	HCs: r = -0.20, p = 0.19<br>pwMS(BZDn): r = -0.08, p = 0.52<br>pwMS(BZDp): r = -0.19, p = 0.43	HCs: r = 0.005, p = 0.97<br>pwMS(BZDn): r = -0.12, p = 0.33<br>pwMS(BZDp): r = -0.23, p = 0.34	HCs: r = -0.01, p = 0.91<br>pwMS(BZDn): r = -0.15, p = 0.22<br>pwMS(BZDp): r = -0.28, p = 0.25

Table S2. Results of Pearson correlation analyses between 1/f slope modulation and cognitive performance scores. This table summarizes the results of exploratory Pearson correlation analyses conducted to investigate the relationship between task-induced 1/f slope modulation and various cognitive test scores. The analyses were performed across all participants to examine whether changes in the 1/f slope are associated with individual differences in cognitive functioning. Cognitive measures include scores from standardized neuropsychological assessments commonly used in multiple sclerosis research including: BVMT-R (Revised Brief Visuospatial Memory Test: to assess visuospatial memory, SDMT (Symbol Digit Modalities Test): to evaluate information processing speed, VGLT (the Dutch version of the California Verbal Learning Test (CVLT-II)): to assess verbal memory, COWAT (Controlled Oral Word Association Test): to measure verbal fluency.
Given the exploratory nature of this analysis, no correction for multiple comparisons was applied. Therefore, the results should be interpreted with caution and considered hypothesis-generating rather than confirmation. For each cognitive measure, the table reports the correlation coefficient (r) and associated p-value.

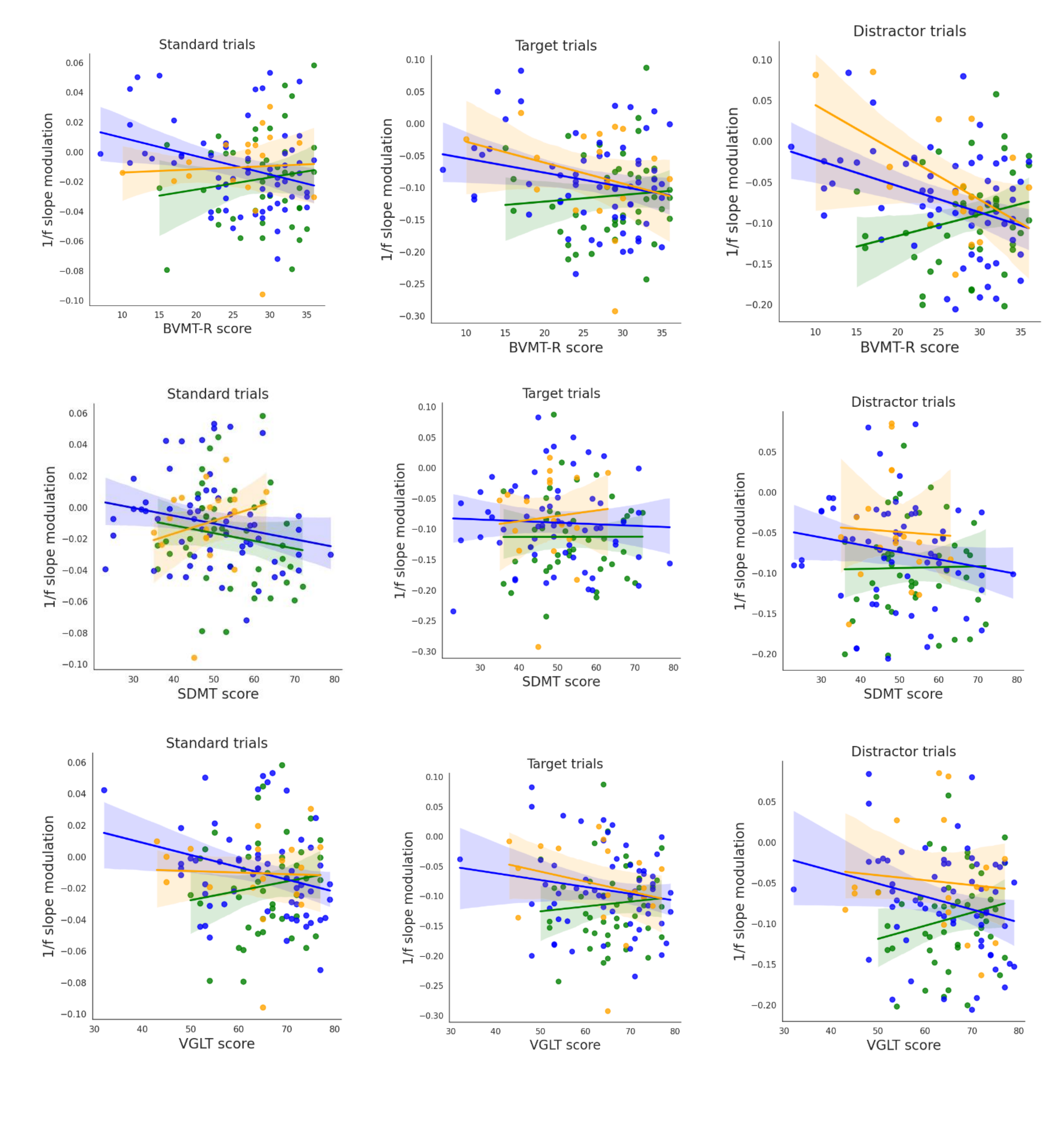
Standard trials
Target trials
Distractor trials
1/f slope modulation
BVMT-R score
Standard trials
Target trials
Distractor trials
1/f slope modulation
SDMT score
Standard trials
Target trials
Distractor trials
1/f slope modulation
VGLT score

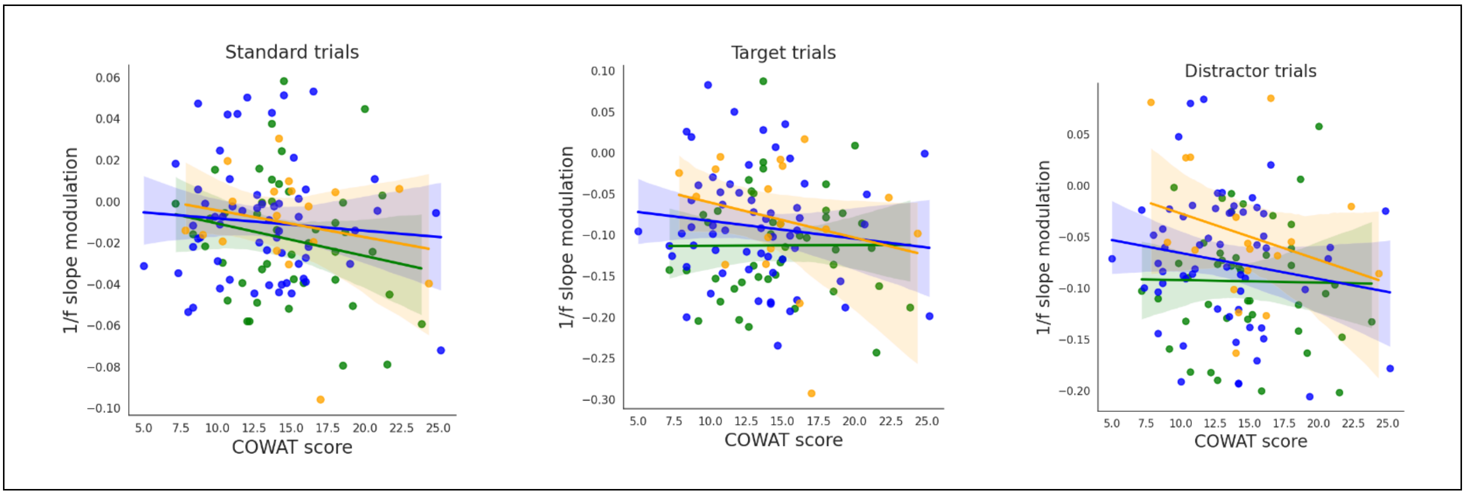

Figure S7. Correlation between 1/f slope modulation and cognitive performance across all subjects. This figure presents scatter plots illustrating the results of exploratory correlation analyses between task-induced 1/f slope modulation and cognitive test scores, aggregated across all participants. Each plot displays individual data points, with **green** representing healthy controls (HCs), **blue** representing people with MS not taking benzodiazepines (pwMS BZDn), and **orange** representing people with MS taking benzodiazepines (pwMS BZDp).The analyses aim to examine whether modulation in the 1/f slope is associated with performance on cognitive tasks. Cognitive scores are derived from standardized assessments, including: BVMT-R (Revised Brief Visuospatial Memory Test: to assess visuospatial memory, SDMT (Symbol Digit Modalities Test): to evaluate information processing speed, VGLT (the Dutch version of the California Verbal Learning Test (CVLT-II)): to assess verbal memory, COWAT (Controlled Oral Word Association Test): to measure verbal fluency. No correction for multiple comparisons was applied due to the exploratory nature of these analyses

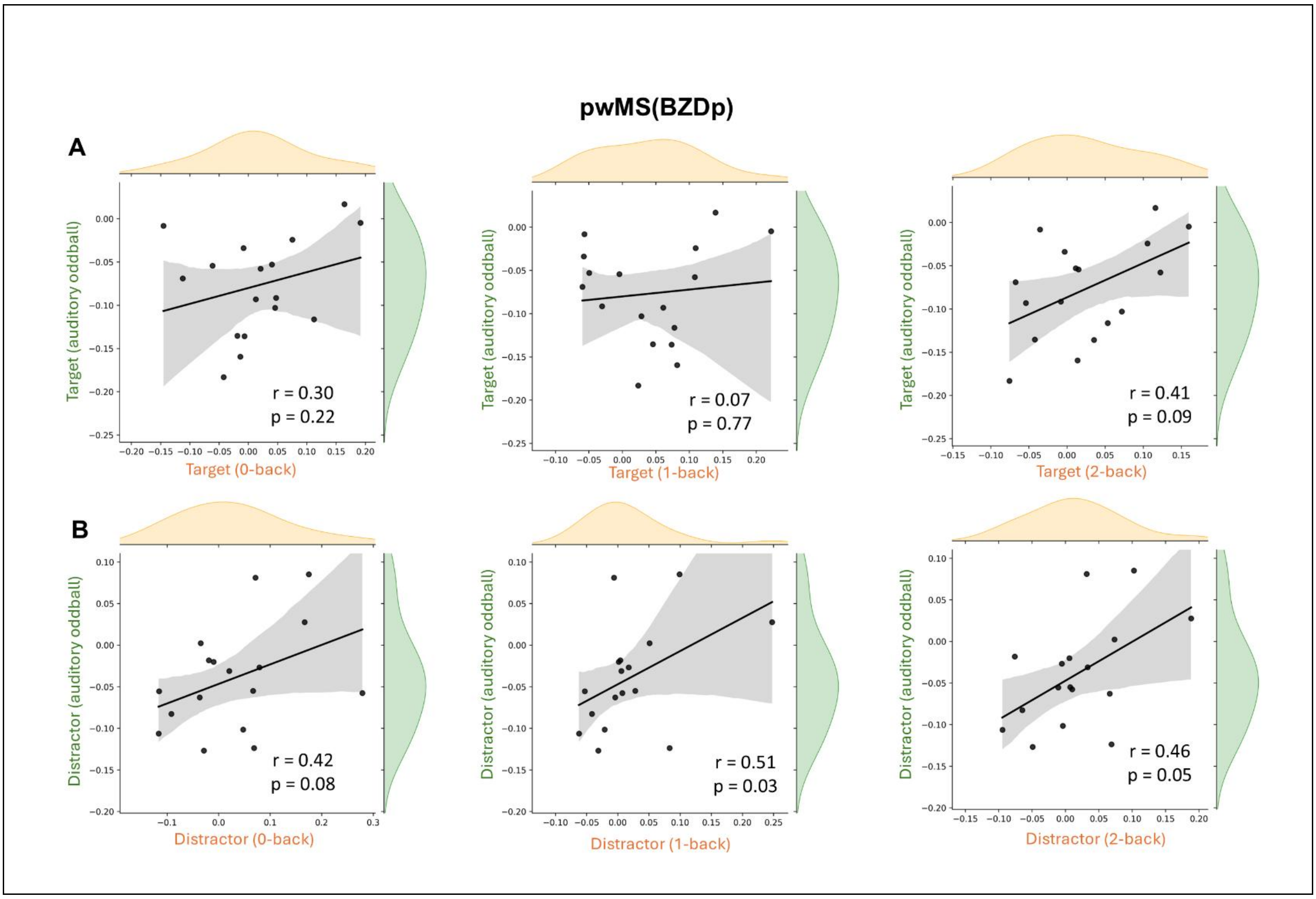

Figure S8. Cross-paradigm correlation of 1/f slope modulation between the auditory oddball and n-back tasks in **pwMS(BZDp).** Scatter plots display the relationship between 1/f slope modulation in the auditory oddball task (y-axis) and the n-back task (x-axis) across three working memory load conditions: 0-back, 1-back, and 2-back. **Panel A:** Correlations for target trials across the two tasks. **Panel B:** Correlations for distractor trials across the two tasks. Each dot represents one participant; shaded bands indicate 95% confidence intervals for the regression line. All Spearman's correlation coefficients (*r*) and corresponding *p*-values reported in each plot.

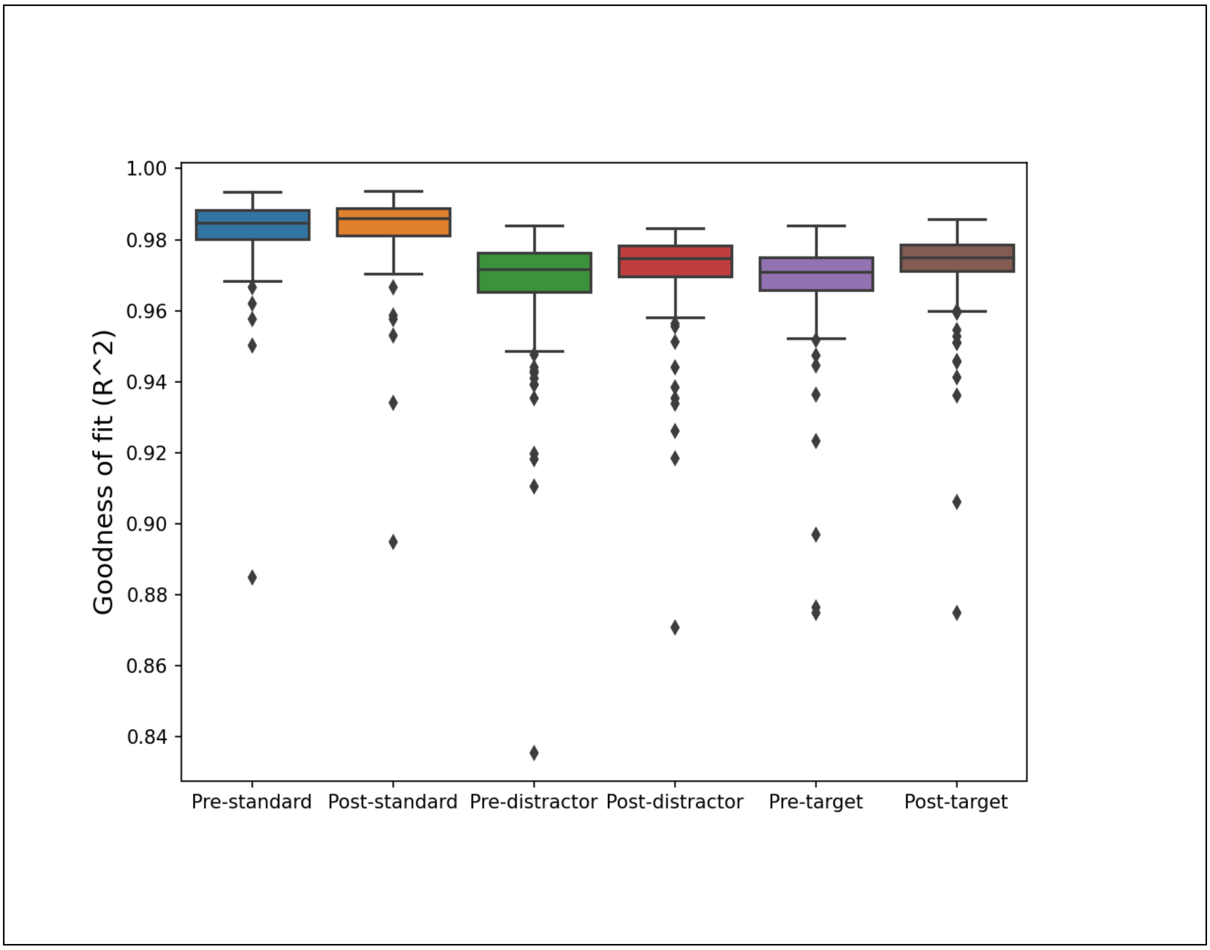

Figure S9. Goodness of Fit. Box plots of the R2 parameter of specparam fitting of power spectrum densities within pre- and post-stimulus time windows for each type of trial for all subjects.

Table S3. Description of the brain parcellation used in this study. The brain was divided into 42 parcels. The table lists each region along with its corresponding center coordinates (X, Y, Z) in MNI coordinates. Abbreviations used in the table include L = left, R = right, VL = Ventrolateral, Lat = Lateral, Inf = Inferior, Sup = Superior, Ant = Anterior, post = Posterior, PFC = prefrontal cortex, and SMC = sensorimotor cortex.

	ROI	X	Y	Z
'ROI 1'	'L Cuneus'	-10.041157	-87.387333	23.264176
'ROI 2'	'R Cuneus'	10.232210	-86.691212	23.173993
'ROI 3'	' L Inf Occ'	-37.404190	-76.715706	-5.013719
'ROI 4'	'R Inf Occ'	37.404705	-76.658476	-5.018754
'ROI 5'	'L Supramarginal'	-52.970271	-9.776851	29.356404
'ROI 6'	'R Supramarginal'	53.692541	-9.833279	29.439441
'ROI 7'	'L Sup Temp'	-53.825784	-22.125427	6.344473
'ROI 8'	'R Sup Temp'	54.828963	-22.395101	6.508962
'ROI 9'	'L Lat SMC'	-38.020122	-26.330033	56.745455
'ROI 10'	'R Lat SMC'	37.960316	-26.063074	57.012851
'ROI 11'	'L Sup Parietal'	-23.068184	-60.800523	53.576483
'ROI 12'	'R Sup Parietal'	23.187349	-60.538696	53.552924
'ROI 13'	'L Middle Occ'	-47.158924	-66.886025	9.105777
'ROI 14'	'R Middle Occ'	46.813637	-67.020472	9.043002
'ROI 15'	'L Sup Occ'	-36.413131	-74.450520	37.353471
'ROI 16'	'R Sup Occ'	36.469556	-74.236308	37.565082
'ROI 17'	'L Ant Temp'	-50.737493	-5.503596	-16.634185
'ROI 18'	'R Ant Temp'	51.018004	-5.085719	-16.800809
'ROI 19'	'L Medial SMC'	-10.698458	-29.081278	64.649915
'ROI 20'	'R Medial SMC'	10.869902	-29.151793	64.945118
'ROI 21'	'L Angular'	-55.576792	-46.995096	33.580583
'ROI 22'	'R Angular'	55.782333	-46.903079	33.708922
'ROI 23'	'L VL PFC'	-42.388358	29.539633	11.893169
'ROI 24'	'R VL PFC'	42.751790	29.874443	12.015973
'ROI 25'	'L Occ pole'	-19.853695	-94.353151	6.547760
'ROI 26'	'R Occ pole'	20.864281	-93.594501	6.603885
'ROI 27'	'L Sup PFC'	-22.890070	10.025135	56.075264

'ROI 28'	'R Sup PFC'	22.978339	10.040136	56.073122
'ROI 29'	'L Sup Dorsal PFC'	-14.715358	36.884207	46.913018
'ROI 30'	'R Sup Dorsal PFC'	14.643759	37.420413	46.667334
'ROI 31'	'L Orbitofrontal'	-31.884408	52.153434	-4.913617
'ROI 32'	'R Orbitofrontal'	32.720804	51.881597	-5.093415
'ROI 33'	'L Post Temp'	-57.681535	-47.586718	-1.401713
'ROI 34'	'R Post Temp'	58.253087	-47.286271	-1.321636
'ROI 35'	'L Inf Dorsal PFC'	-20.767574	54.692649	19.758249
'ROI 36'	'R Inf Dorsal PFC'	20.907743	54.983629	19.797617
'ROI 37'	'Medial PFC'	0.051331	44.666672	10.474446
'ROI 38'	'Post Precuneus'	1.416289	-67.151331	36.146836
'ROI 39'	' Posterior Cingulate Cortex'	1.149822	-48.563740	23.829060
'ROI 40'	'Ant Precuneus'	0.889045	-55.058007	48.993262
'ROI 41'	'L Inf Parietal'	-44.714910	-59.283973	23.670114
'ROI42'	'R Inf Parietal'	47.447109	-58.660973	23.670013
